# Keto-Polyethylenes with Controlled Crystallinity and Materials Properties from Catalytic Ethylene–CO–Norbornene Terpolymerization

**DOI:** 10.1021/acs.macromol.3c02309

**Published:** 2024-01-12

**Authors:** Fabio De Stefano, Maximilian Baur, Claudio De Rosa, Stefan Mecking

**Affiliations:** † Chair of Chemical Materials Science, Department of Chemistry, University of Konstanz, Konstanz 78464, Germany; ‡ Dipartimento di Scienze Chimiche, Università di Napoli Federico II, Complesso Monte S. Angelo, Via Cintia, Napoli I-80126, Italy

## Abstract

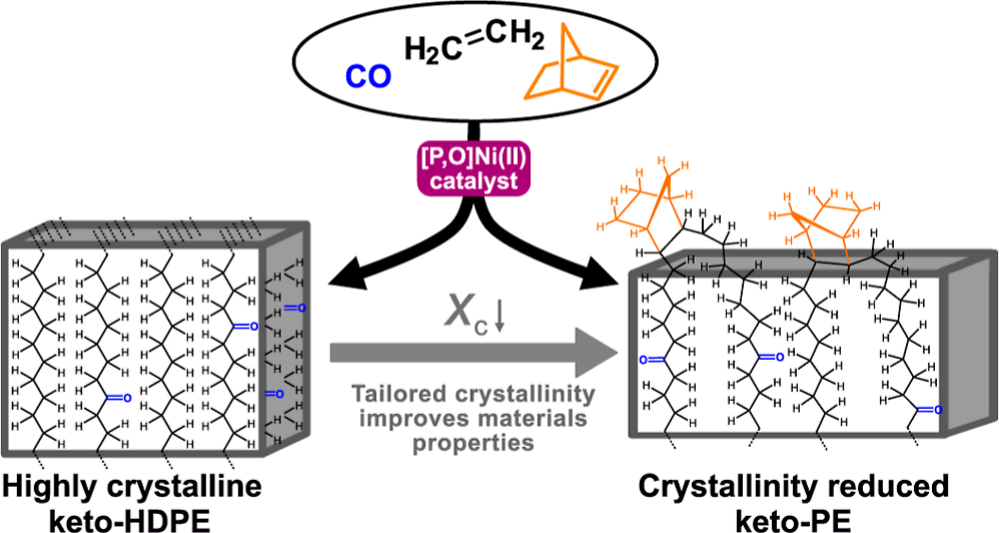

Recent advances in Ni(II) catalyzed, nonalternating catalytic copolymerization of ethylene with carbon monoxide (CO) enable the synthesis of in-chain keto-functionalized polyethylenes (keto-PEs) with high-density polyethylene-like materials properties. Addition of norbornene as a bulky, noncrystallizable comonomer during catalytic polymerization allows tuning of the crystallinity in these keto-PE materials by randomly incorporated norbornene units in the polymer chain, while molecular weights are not adversely affected. Such crystallinity-reduced keto-PEs are characterized as softer materials with better ductility and may therefore be more suited for, e.g., potential film applications.

## Introduction

Polyethylene (PE), representative of a semicrystalline polymer, exhibits a flexible molecular structure and is distinguished by a high crystallinity, yielding a strong and ductile material at room temperature. Since several applications require improved impact properties or enhanced transparency and deformability, precise control over materials properties by tailored modifications of the molecular structure or molecular weight distribution can further expand the range of PE’s potential applications. Branching is one common strategy to modify the molecular structure and, therefore, crystallinity and crystallization behavior.^1−3^ For example, low-density polyethylene (LDPE) from free radical polymerization exhibits a highly branched molecular structure compared to high-density polyethylene (HDPE) from transition metal-catalyzed ethylene polymerization, which consists of linear hydrocarbon chains devoid of branches.^2^ These different microstructures impact crystallization of the hydrocarbon chains, resulting in either crystalline, therefore more rigid, HDPE materials or soft LDPE materials with low crystallinity due to inhibited –CH_2_– alignment in folded chain crystallites.^2,4^ However, free-radical polymerization offers only limited control over branch formation.^5^ A more controlled strategy to influence the crystallinity and properties of PE materials is the copolymerization of ethylene with suitable comonomers.^6−13^ In particular, the addition of a small amount of randomly distributed, noncrystallizable units into the PE main chain, similar to the branches found in LDPE, can alter the crystallization ability and decrease its melting temperature, as well as crystallinity. Common examples include ethylene copolymers with linear α-olefins to yield linear low-density polyethylene (LLDPE),^6−15^ which is applied extensively in, e.g., packaging films for commodities or structural components.

Especially, the class of cyclic olefin copolymers (COCs) employing norbornene as a cyclic, noncrystallizable unit has gained particular attention due to their versatility. They possess tunable properties ranging from highly crystalline solids to thermoplastic elastomers, depending on the concentration and distribution of norbornene in the polymer chain.^16−19^ Ethylene–norbornene copolymers were first synthesized using methylalumoxane-activated metallocene systems.^20−22^ Later, titanium-based nonmetallocene and other constrained-geometry group IV catalysts were described in the synthesis of these materials.^17,23−25^ Late transition metal catalysts (based on nickel and palladium), which are very active in homopolymerization of norbornene, have also been employed in copolymerization with ethylene, yielding copolymers with variable contents of norbornene and its functionalized derivatives.^26−31^ However, the majority of these literature-reported COCs exclusively focuses on amorphous copolymers with high norbornene contents (>30 mol %), which display high glass transition temperatures and properties that differ completely from those of PE.

Materials properties of PE beyond crystallinity can also be influenced by the introduction of functional groups to the otherwise highly apolar and hydrophobic polymer. Such polar functional groups in the hydrocarbon-based polyethylene chain increase its polarity and therefore enhance barrier properties, adhesion to or compatibility with polar materials.^32−34^ This is commonly achieved by either postpolymerization C–H oxidation^33,35^ or by copolymerization of ethylene with polar vinyl monomers.^36−43^ In contrast to vinyl comonomers, the copolymerization of ethylene with carbon monoxide (CO) can yield keto groups directly in the polyethylene backbone. At low functional group densities, these in-chain keto groups can be included into the polyethylene crystallites.^44−46^ Additionally, these in-chain keto groups impart photodegradability to the polymer, which offers a potential alleviation of the environmental impacts of mismanaged PE waste.^47−52^ In particular, linear HDPE-like materials from catalytic copolymerization of ethylene and CO have been long sought for and are particularly challenging to access due to preferred insertion of CO over the ethylene comonomer, often leading to the formation of alternating polyketone (*T*_m_ > 220 °C) instead.^53−55^ Such materials have only recently been enabled by nonalternating copolymerization of ethylene and CO.^52,56−60^ Advanced neutral phosphinophenolate Ni(II) catalysts^61−65^ have been particularly suitable for this direct catalytic copolymerization of ethylene and CO, yielding photodegradable keto-PE materials with high molecular weights (up to *M*_w_ 400 000 g mol^–1^) and virtually uncompromised HDPE-like properties.^42,51,66,67^

However, these keto-PE materials are highly crystalline, which may limit their possible applications if softer materials are required. We now report the use of norbornene as a noncrystallizable, second comonomer in nonalternating Ni(II)-catalyzed ethylene-CO copolymerization. This enables control over crystallinity in the obtained keto-PEs and can improve their physical and mechanical materials properties, while not adversely affecting molecular weights of the obtained terpolymers, unlike in ethylene-CO acrylate terpolymerization.^42^

## Results and Discussion

**Scheme 1:**
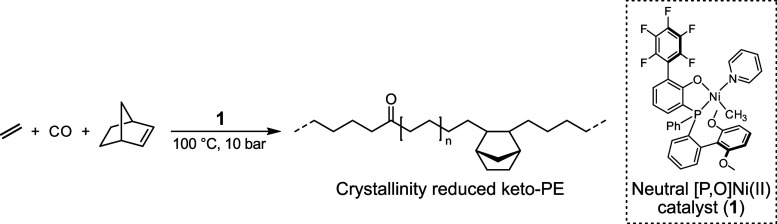
Terpolymerization of Ethylene with CO and Norbornene as a Bulky Comonomer to Crystallinity-Reduced Keto-Functionalized Polyethylene Catalyzed by a State-of-the-Art Phosphinophenolate Ni(II) Catalyst (**1**)

The exposure of a state-of-the-art neutral phosphinophenolate Ni(II) catalyst **1**, previously reported for efficient co- and terpolymerizations of ethylene and CO,^42,51,67^ to a mixed feed of gaseous ethylene and CO (10 atm total pressure) with a low ratio of CO (0.8%) in the presence of norbornene at different concentrations resulted in the formation of solid PE-like polymers (Scheme 1 and Table 1).

**Table 1:** Results of the Catalytic Terpolymerization of Ethylene with CO and Norbornene (NB)

entry no.	CO/C_2_H_4_ feed^a^[ %]	Conc. NB^b^ [mol L^–^^1^]	yield [g]	Χ (CO)^c^ [mol %]	Χ (NB)^d^ [mol %]	*M*_n_ (*M*_w_/*M*_n_)^f^ [10^3^ g mol^–^^1^]	*T*_m_ [°C]^e^
1		0.1	3.3		0.7	84 (1.7)	124
2	0.8		2.15	1.3		65 (1.6)	136
3	0.8	0.1	1.37	0.9 (1.1)	0.5	43 (1.7)	126
4	0.8	0.15	1.0	0.7 (1.1)	0.7	34 (1.7)	126
5	0.8	0.2	1.06	0.7 (0.7)	1.1	40 (1.7)	124
6^g^	0.8	0.2	1.05	1.0 (1.7)	1.2 (1.1)	50 (1.6)	122
7	0.8	0.3	1.05	0.9 (1.4)	1.4	38 (1.8)	124
8	0.8	0.4	1.07	0.7 (1.3)	1.3	41 (1.8)	122
9	0.8	0.5	0.67	1.1 (1.0)	3.3	29 (2.0)	97/124

a Polymerization conditions: 10 μmol precat. **1**, 10 atm, 0.8% CO in a C_2_H_4_ feed, 100 °C, 1000 rpm, 75 min, 100 mL toluene. Ratio of CO in an ethylene-CO gas feed.

b Concentration of norbornene in the initial reaction solution.

c Determined by ATR-IR spectroscopy (cf. Supporting Information for details). In brackets: Incorporation determined by ^1^H NMR spectroscopy by integration of the ^1^H signals of α-carbonyl C*H*_2_ (CO) in relation to the overall integral.

d Determined by ^1^H NMR spectroscopy by integration of ^1^H signals of norbornene H1 and H4 protons at 2.00 ppm. In brackets: Incorporation determined by quantitative ^13^C NMR spectroscopy.

e Determined by SEC in 1,2-dichlorobenzene at 160 °C (1.0 mL min^–1^) via linear calibration with narrow PE standards.

f Determined by DSC, second heating cycle (10 K min^–1^).

g ^13^CO employed as a comonomer.

**Figure 1:**
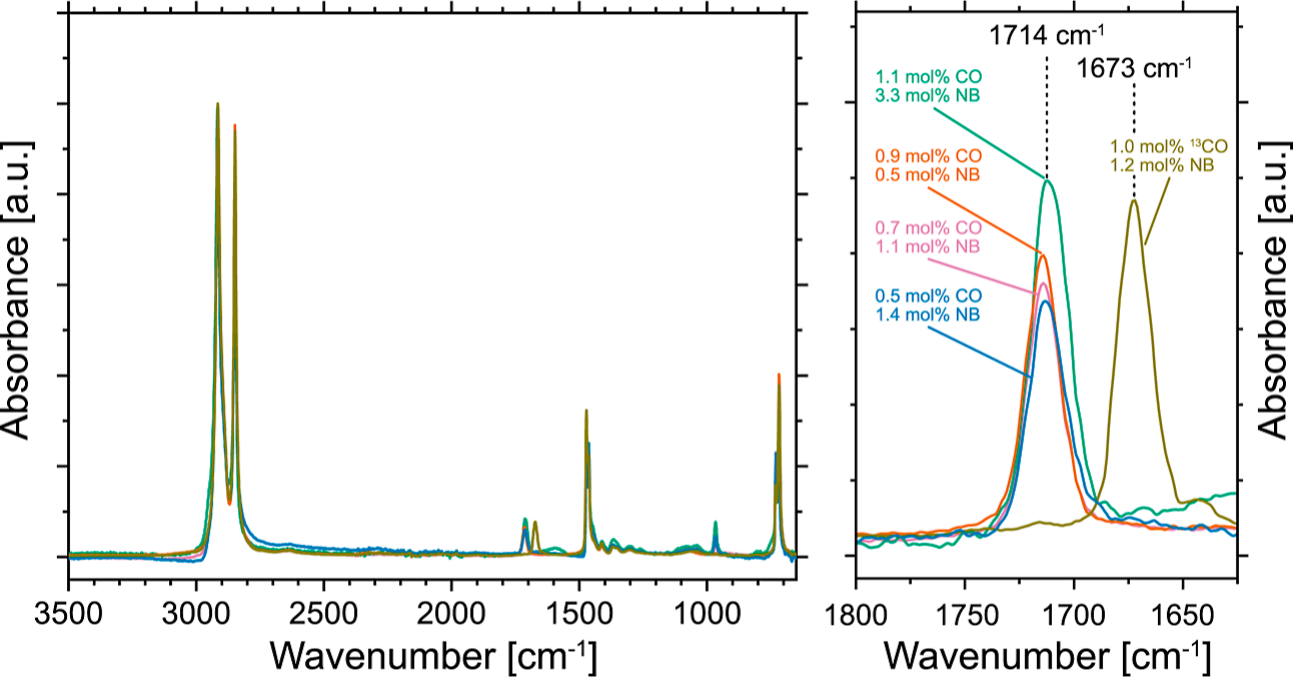
ATR-IR spectra (left) with details of the carbonyl region (right) of ethylene-norbornene-CO terpolymers. Carbonyl absorption bands at 1714 cm^–1^ (1673 cm^–1^ for ^13^CO-labeled samples) show the mainly isolated nature of in-chain carbonyl groups.

**Figure 2:**
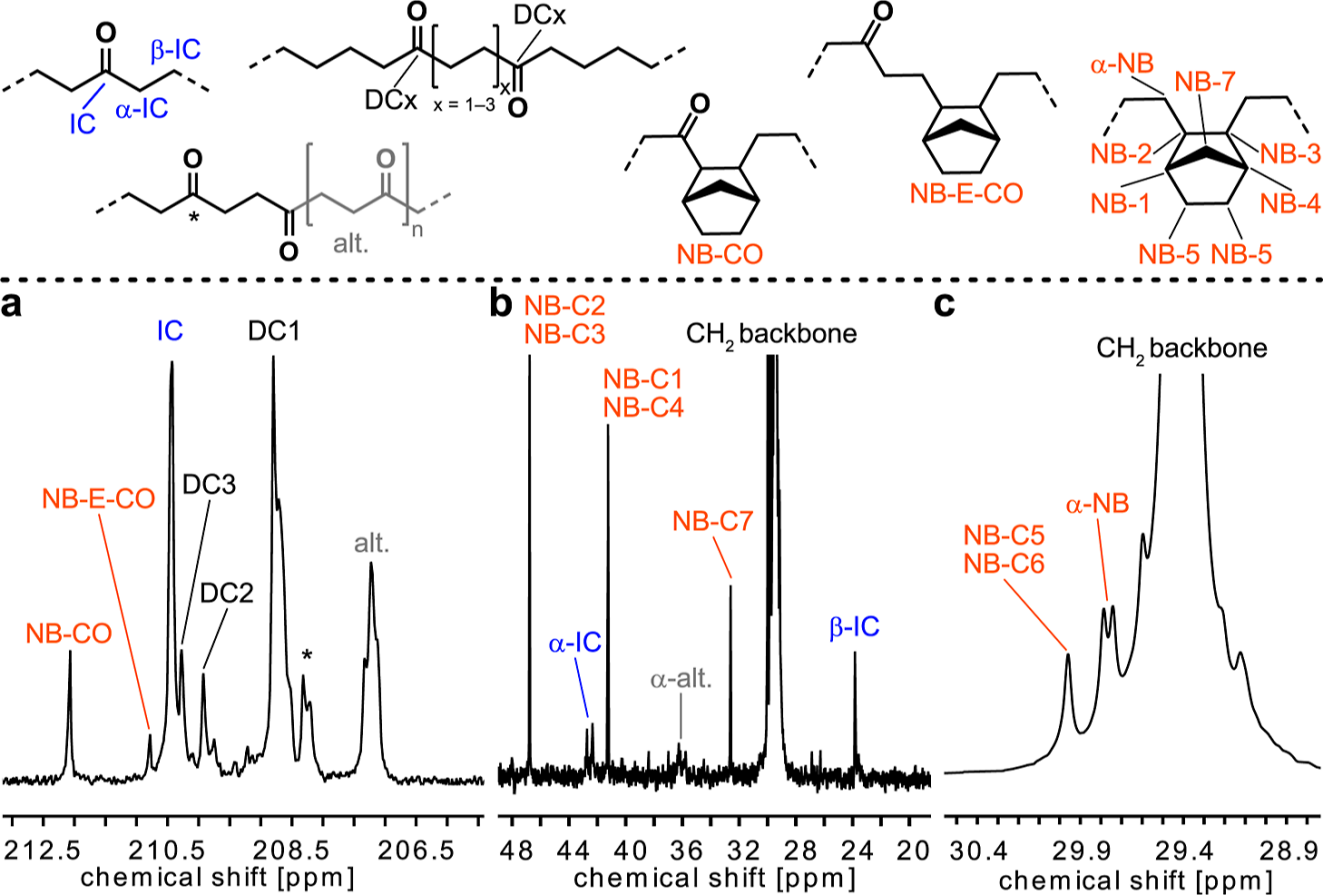
^13^C NMR spectra (101 MHz, 383 K, C_2_D_2_Cl_4_) showing the carbonyl microstructure (**a**) and linearly incorporated isolated norbornene units (**b** and **c**) in a ^13^CO-labeled, crystallinity-reduced keto-PE obtained from terpolymerization with complex **1**.

Polymerization activities and polymer yields are reduced by the combined presence of both comonomers norbornene and carbon monoxide compared to the respective ethylene copolymerization employing only one respective comonomer (Table 1, entries 1 and 2). However, the decrease in the yield and activity is much less pronounced compared to previously reported terpolymerizations of ethylene, CO, and acrylates.^42,68^ Furthermore, polymerization yields are not largely influenced by the variation of norbornene in the initial reaction mixture. The analysis of the obtained polymers by attenuated total reflectance (ATR)-IR spectroscopy revealed the incorporation of predominantly isolated in-chain keto groups by the presence of an C=O absorption peak at 1715 cm^–1^, which is characteristic for the latter (Figure 1).^45,50,69^ Quantitative analysis of IR spectra allowed calculation of the C=O incorporation ratios, which are around the target value of approximately 1 mol % (0.7–1.1 mol %). The analysis by ^1^H and ^13^C NMR spectroscopy confirmed the presence of in-chain keto groups as well as their incorporation in a largely isolated fashion (Figures 2 and S4–S10). For enhanced sensitivity in ^13^C NMR spectroscopy, ^13^CO instead of ^12^CO was employed as a comonomer to conveniently introduce isotopic labeling in a representative copolymer (Table 1, entry 6). The carbonyl microstructure was found to be comparable to our previously reported keto-PEs^46,51,67^ with the majority of in-chain carbonyl groups incorporated as isolated units in the polymer backbone, as expected from observations by IR spectroscopy (Figure 2a).

Due to the lack of IR-sensitive functional groups in norbornene, incorporation of the second comonomer was visible only in ^1^H and ^13^C NMR spectroscopy, which was also used for simultaneous quantification of the norbornene content in the obtained terpolymers. Norbornene contents in a range of 0.5–3.3 mol % could be obtained, which were found to be controllable by the initial norbornene concentration applied in the reaction mixture (cf. Table 1). Ethylene incorporation is favored over norbornene incorporation and in fact, only a small amount of the initially present norbornene is reacted, corresponding to near steady-state conditions of norbornene monomer concentration (<10% conversion as determined from the composition of the initial reaction mixture, and the amount and composition of polymer formed). Note that the other monomers, ethylene and CO, are replenished by the automated feed system. Thorough investigation by 1D and 2D NMR spectroscopy allowed for a complete assignment of all signals observed in NMR spectroscopy (cf. Figure 2 and Supporting Information).

This revealed the incorporation of norbornene in an exclusively isolated fashion, and neither alternating nor block-like motifs could be observed. Indeed, the observed polymer microstructure was in line with previously reported random ethylene-norbornene copolymers with low norbornene contents.^70−72^ The occurrence of additional polynorbornene resonances was not related to the formed ethylene-norbornene-CO terpolymer but was referred to a small and variable fraction of polynorbornene formation by a ROMP mechanism upon the exposure of norbornene to the Ni catalyst or impurities in the setup at the elevated polymerization temperatures.^73^ These amorphous polynorbornene fractions could be removed from the desired terpolymer by washing with toluene without affecting any materials properties of the crystallinity-reduced keto-PEs (cf. Supporting Information) (Figure S17 and Table S3). In addition to the characteristic norbornene resonances, the observation of new carbonyl signals in ^13^C NMR spectroscopy on ^13^CO-labeled samples showed the presence of a carbonyl group adjacent to a norbornene unit, as well as a carbonyl group separated by likely one −(C_2_H_4_)– unit from ethylene insertion between a respective CO and a norbornene insertion event (Figure 2a). Nevertheless, no preference for promoted insertion of either comonomer after the other could be observed, similar to previous reports on ethylene-CO terpolymerization with vinylic monomers.^42,68^ Contrary to previously reported ethylene co- and terpolymerizations with other vinyl monomers,^42,62,68,74^ only end groups from expected chain termination by β-H elimination after an ethylene incorporation and no enhanced chain termination by the presence of norbornene were observable. In fact, terpolymer molecular weights are only slightly lowered by the presence of norbornene as a second comonomer and are accessible in a similar range compared to the keto-PE without norbornene (cf. Table 1, entry 2 vs 6, and Figure S14).

**Figure 3:**
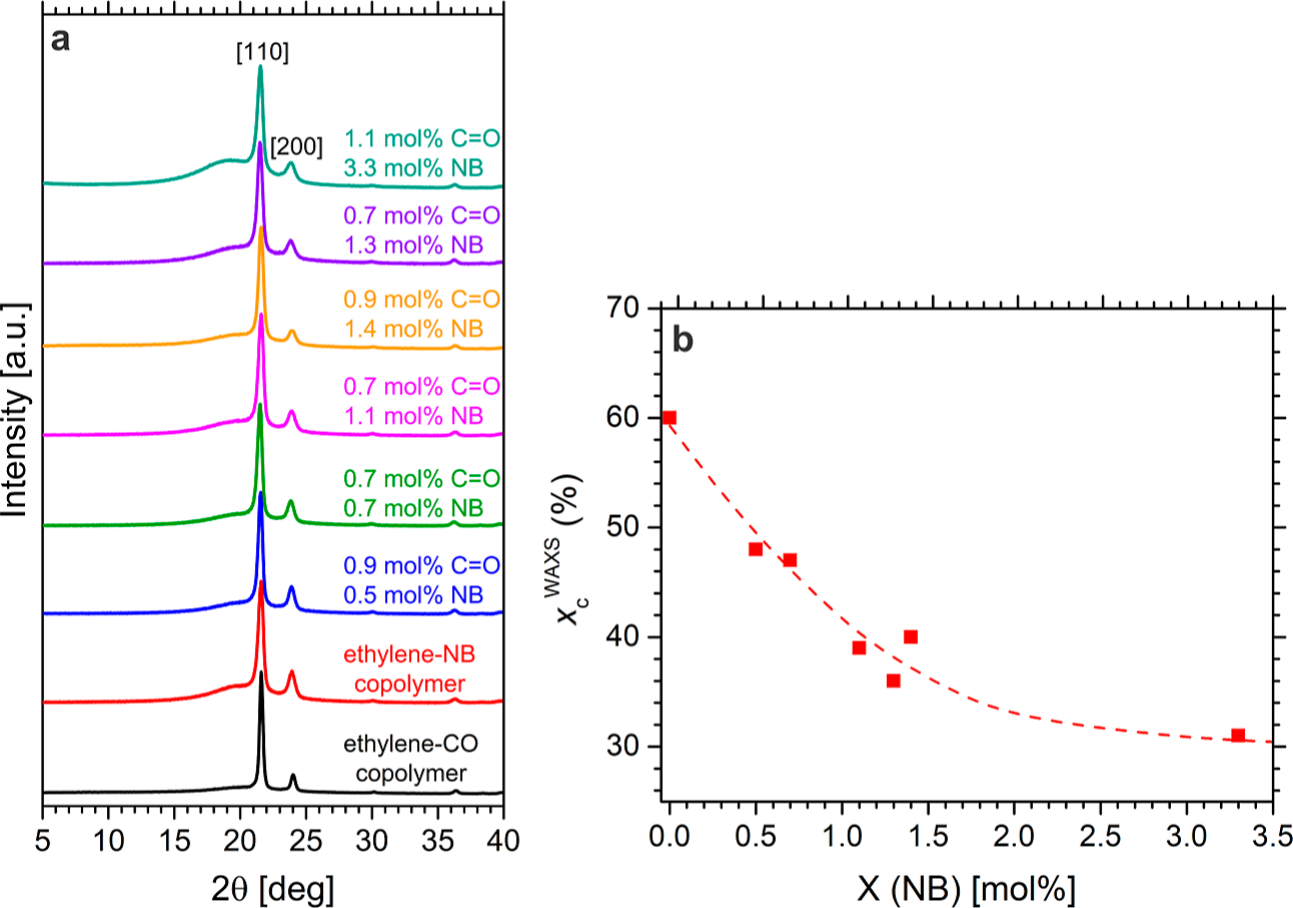
WAXS traces of melt-crystallized ethylene–CO–norbornene terpolymers of the indicated CO and NB content (**a**). Values of the degree of crystallinity (*x*_c_^WAXS^) of the melt-crystallized samples as a function of the NB content (**b**). In (**a**), the diffraction profiles of ethylene-CO and ethylene-norbornene copolymer samples prepared with the same catalyst are also reported. The 110 and 200 reflections of the orthorhombic form of PE at 2θ ≈ 21.4° and 23.8°, respectively, are indicated. Traces are vertically shifted for clarity. Note that the interpolation line in (**b**) is just a guide for the eye.

Wide angle X-ray powder diffraction (WAXS) profiles collected on melt-crystallized samples indicated that all ethylene–CO–norbornene terpolymers crystallize in an orthorhombic solid-state structure characteristic of PE (Figure 3a). The incorporation of the small amounts of carbon monoxide and norbornene, even in the terpolymer with the highest norbornene content of 3.3 mol % (Table 1, entry 9), does not have a significant effect on the crystalline packing of the polyethylene chains in the range of explored comonomer contents. Nevertheless, a significant reduction of crystallinity with comonomer incorporation was observed. In particular, the degree of crystallinity, evaluated from WAXS diffraction profiles (cf. Supporting Information) (Figure S15), is rather high [*xc*WAXS = 60%, *xc*DSC = 63%] in the neat keto-PE containing only ethylene and CO comonomers and gradually decreases in the terpolymers with increasing norbornene content, from nearly 48% of sample 3 with 0.5 mol % NB to about 31% of sample 9 with 3.3 mol % NB (Figure 3b). The influence of the presence of norbornene counits on PE crystallinity has been thoroughly investigated by Alamo et al.^13^ These studies included several samples of random ethylene-norbornene copolymers in a compositional range similar to the terpolymers investigated in this work (1–5 mol % NB). Alamo et al. further demonstrated that, for low comonomer concentrations, the impact of norbornene on the PE crystallinity is virtually identical to those reported for ethylene–1-alkene (1-butene, 1-hexene, 1-octene) copolymers.^13^ Our results are consistent with those reported in ref (13), again confirming that the presence of in-chain carbonyl groups does not affect crystallinity and, hence, the decrease in crystallinity is solely due to the incorporated norbornene. WAXS data also confirm the predominantly isolated nature of the in-chain carbonyl groups as evident by the absence of the (110) reflection 2θ ≈ 22.5° related to alternating polyketone crystals (Figure 3a).^75^

**Figure 4:**
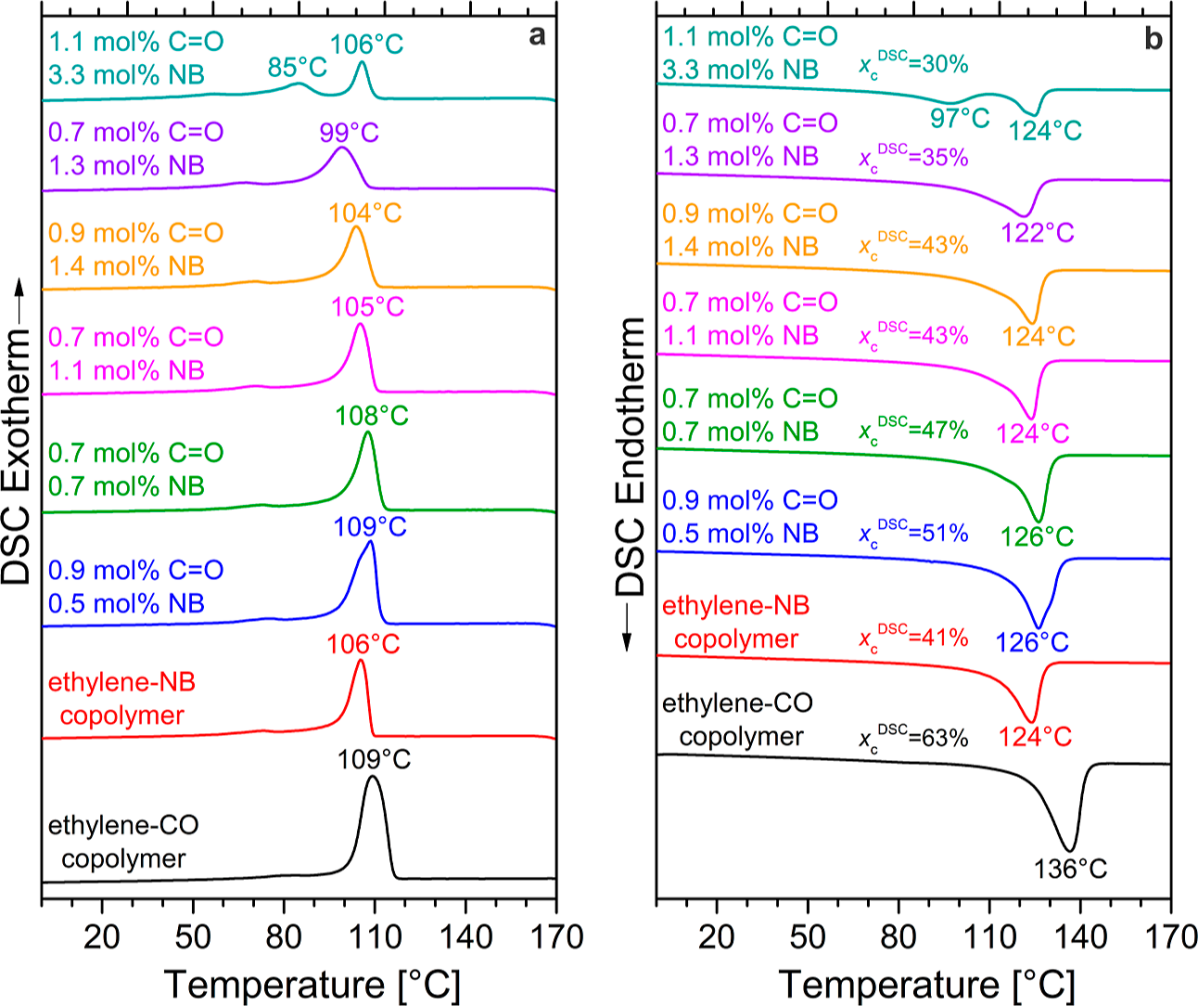
DSC thermograms of samples reported in Table 1 recorded at 10 °C min^–1^ during cooling from the melt (**a**) and successive heating (**b**). In (**b**), values of the degree of crystallinity (*x*_c_^DSC^) of the melt-crystallized samples, evaluated as reported in the Supporting Information, are also given.

DSC thermograms (Figure 4) clearly confirm the incorporation of norbornene into all synthesized terpolymer samples (Figure 4), in line with NMR spectroscopic analysis. In fact, crystallization (Figure 4a) and melting (Figure 4b) points gradually decrease as the NB contents increase. However, it is worth noting that both melting and crystallization temperatures are only mildly affected by the presence of the two comonomers retaining relatively high values, only slightly lower than those of HDPE, even for norbornene contents above 3 mol %. Such thermal properties are a particular prerequisite for the potential processing of the obtained materials employing established methods. Only for the sample with the highest concentration of NB units (1.1 mol % CO and 3.3 mol % NB), the DSC curves show two crystallization peaks (Figure 4a) and two melting peaks (Figure 4b), which can be attributed to a slightly heterogeneous microstructure with consequent crystallization and successive melting at high temperatures of chain segments poorer in NB units and crystallization and melting at the lower temperature of chain segments richer in NB units.

**Figure 5:**
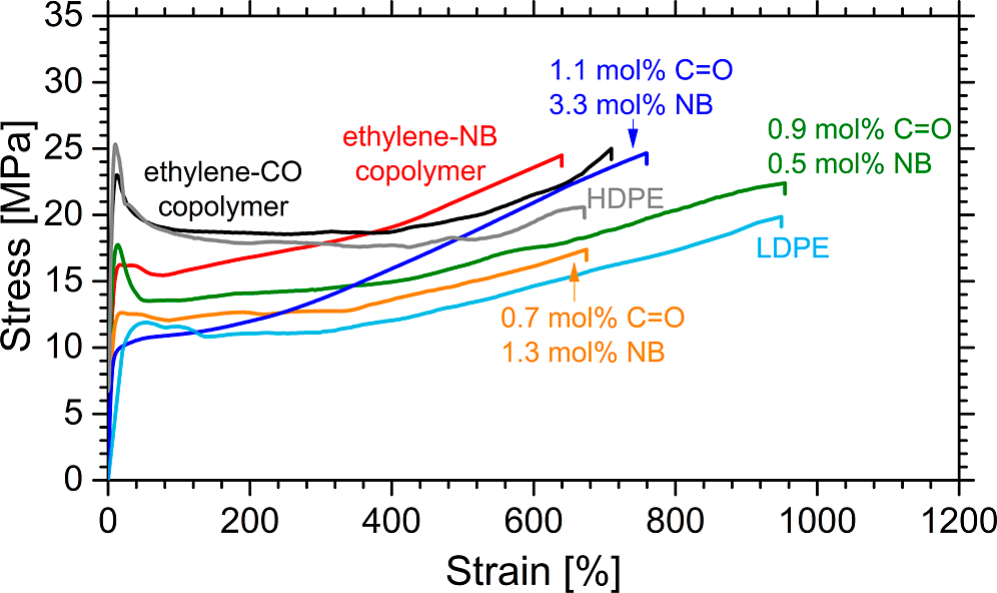
Stress–strain curves of selected samples: reference ethylene-CO and ethylene-norbornene copolymers (black and red, respectively), and different ethylene–CO–norbornene terpolymers with a comonomer content of 0.9 mol % CO and 0.5 mol % NB (green), 0.7 mol % CO and 1.3 mol % NB (blue), and 1.1 mol % CO and 3.3 mol % NB (orange). Stress–strain curves of commercial HDPE and LDPE samples are also reported for comparison.

The mechanical properties of all ethylene–CO–norbornene terpolymer samples were studied on compression-molded films (Figure 5). All terpolymers, as well as ethylene-CO (keto-PE) and ethylene-NB reference copolymers, exhibit deformation with necking and are characterized by remarkable strength and deformability with high values of strain at break, higher than 600–800% (Figures 5, S16, and Table S2).

**Figure 6:**
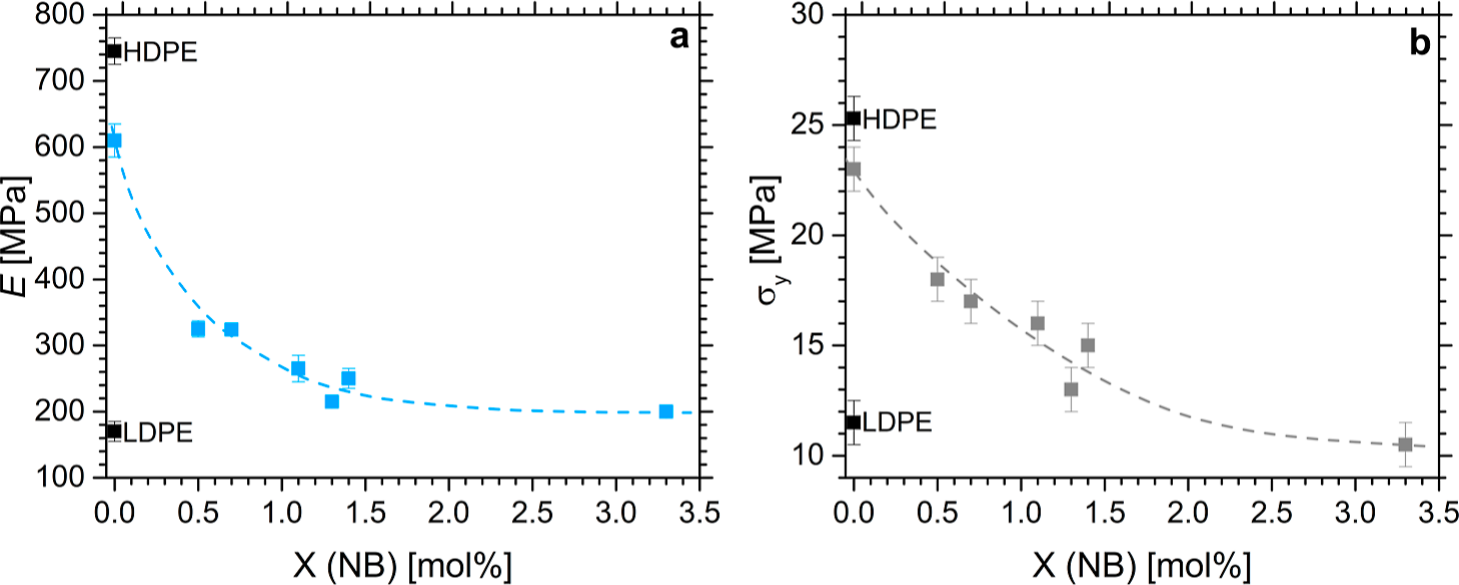
Values of Young’s modulus (*E*) (**a**) and stress at yield (σ_*y*_) (**b**) evaluated from the stress–strain curves recorded on compression-molded films of all synthesized samples as a function of the norbornene content. Note that the interpolation lines are just guides for the eye.

The copresence of carbon monoxide and norbornene resulted in a moderate enhancement of ductility in terpolymers compared with keto-PE (Figures 5, S16, and Table S2). Moreover, the simultaneous incorporation of both defects in the polyethylene backbone results in a decrease in the stress at yield (σ_*y*_) (Figure 6a) and Young’s modulus (*E*) (Figure 6b) that both progressively decrease with increasing norbornene content, in accordance with the reduction in crystallinity. In particular, *E* ranges from ≈610 MPa for the neat keto-PE to ≈200 MPa in the case of the terpolymer with the highest NB content (Figure 6a and Table S2). Considering the typical values of HDPE and LDPE Young’s moduli (≈900 and ≈240 MPa, respectively),^2^ our data indicate that the precise norbornene incorporation enables a controlled modification of stiffness and yield stress while keeping high deformability to achieve a broad spectrum of material properties.

## Conclusions

The addition of norbornene to the established Ni-catalyzed nonalternating copolymerization of ethylene and CO can yield terpolymers with isolated norbornene units in the polymer chain. Contrary to the previously reported terpolymerization of ethylene-CO and polar vinyl monomers,^42^ no involvement of norbornene in enhanced chain transfer rates could be detected and molecular weights are largely retained compared to neat ethylene-CO copolymerization. The bulky norbornene groups act as noncrystallizable units in these high molecular weight keto-PEs and degrees of crystallinity are substantially reduced compared to neat, highly crystalline keto-PE. Nevertheless, crystallinity-reduced keto-PEs retain the basic thermal and crystallization behavior of polyethylene, which allows for melt processing. Such melt-processed, crystallinity-reduced keto-PEs showed improved ductility and lower stress at yield in tensile tests. Therefore, the inclusion of bulky norbornene units as noncrystallizable units might be used as a straightforward tool to tailor materials properties of otherwise highly crystalline and thus mostly rigid keto-HDPEs. This can enable, for example, film applications.

## Supporting Information Available

Experimental procedures and polymer characterization data (PDF)
