# Supplementary material for: Keto-Polyethylenes with Controlled Crystallinity and Materials Properties from Catalytic Ethylene–CO–Norbornene Terpolymerization
Source: Macromolecules. 2024 Jan 12;57(3):1072–9. doi: 10.1021/acs.macromol.3c02309 (PMC10867887; doi:10.1021/acs.macromol.3c02309)
Supplement: Supplementary file 1 — ma3c02309_si_001.pdf [file ma3c02309_si_001.pdf]

Supporting Information

for

Keto-polyethylenes with controlled crystallinity and materials properties from catalytic ethylene-CO-norbornene terpolymerization

Fabio De Stefano[‡,a,b], *Maximilian Baur*[‡,a], *Claudio De Rosa*[b] *and Stefan Mecking*[a,*]

[a]Chair of Chemical Materials Science, Department of Chemistry, University of Konstanz, 78457 Konstanz, Germany.

[b]Dipartimento di Scienze Chimiche, Università di Napoli Federico II, Complesso Monte S. Angelo, Via Cintia, I-80126 Napoli, Italy.

Contents

1. General considerations........ 2
1.1.1 Solvents and reagents........ 2
1.1.2 Analytical methods and techniques........ 2
1.2 General polymerization procedure........ 5
2. Polymer characterization........ 6
2.1 Supplementary IR spectra of polymers........ 6
2.2 Supplementary NMR spectra of polymers........ 7
2.3 SEC traces of polymers........ 13
2.4 Thermal properties from differential scanning calorimetry (DSC)........ 14
2.5 WAXS data of polymers and crystallinity evaluation........ 15
2.6 Tensile properties of crystallinity reduced keto-PEs........ 16
2.7 Removal of polynorbornene impurities........ 17
3. Supporting References........ 19

1. General considerations

Unless noted otherwise, all manipulations of air and moisture sensitive materials were carried out under an inert gas atmosphere using standard glovebox and Schlenk techniques.

1.1.1 Solvents and reagents

Solvents were dried and degassed using standard laboratory techniques. Toluene was dried over molecular sieves (3 Å) and degassed by passing through columns with alumina and BASF R3-11 catalyst. The catalyst precursor **1** was prepared according to a reported procedure.[1] Norbornene was purchased from Sigma Aldrich. All other commercially available reagents were supplied by Sigma Aldrich, Acros, ABCR or Activate Scientific. 1,1,2,2-tetrachloroethane-d_2 was purchased from Eurisotop. Ethylene of grade 3.5 and carbon monoxide of grade 4.7 were supplied by Air Liquide and used as received. ^{13}CO with an isotopic purity of > 99 % was purchased from EurisoTop

1.1.2 Analytical methods and techniques

Polymer NMR spectra were acquired in 1,1,2,2-tetrachloroethane-d_2 at 110 °C and were recorded on a Bruker Avance III HD 400 (^{1}H: 400 MHz, ^{13}C: 101 MHz). ^{1}H chemical shifts were referenced to the residual $C_2H_2Cl_4$: 6.00 ppm. ^{13}C chemical shifts were referenced to the carbon signal of the deuterated solvent ($C_2D_2Cl_4$: 73.78 ppm).

Size exclusion chromatography (SEC) was performed on a PolymerChar GPC-IR instrument equipped with an integrated four-capillary viscometer and an IR4 dual wavelength infrared detector (selective for methylene and methyl groups) on PSS Polefin Linear XL columns (3 × 30 cm) and with an additional guard column at 160 °C in 1,2-dichlorobenzene at 1.0 mL min^{-1} flow rate. Linear calibration with narrow polyethylene standards was employed. The raw data was evaluated with PSS WinGPC UniChrom software.

Differential scanning calorimetry (DSC) was performed on a Netzsch DSC 204 F1 with a bicyclic temperature program and heating/cooling rates of 10 K min^{-1}. For the measurements, the polymers were weighed into sealed 40 µL aluminum pans. The degrees of crystallinity x_c^{DSC} were evaluated as: $x_c^{DSC} = \Delta H_m / \Delta H_m^0$ where ΔH_m^0 = 297 J/g is the thermodynamic melting enthalpy of 100% crystalline PE.[2]

X-ray diffraction patterns were recorded at room temperature with Ni filtered Cu Kα radiation (wavelength λ=0.15418 nm). The powder diffraction profiles were obtained with an automatic PANalytical Empyrean diffractometer operating in the reflection geometry with continuous scans of the 2θ angle and scanning rate of 0.02 degree/s. WAXS measurements were

recorded on polymer samples obtained from controlled melt crystallization at 10 K min^{-1}. The degrees of crystallinity (x_c^{WAXS}) of all melt-crystallized samples were determined from the X-ray powder diffraction profiles of Figure 3, **a** as the ratio between the area of the crystalline diffraction peaks (A_c) and the area of the whole diffraction profiles (A_t), $x_c = (A_c/A_t)\times 100$. The area of the crystalline diffraction peaks A_c was evaluated by subtracting the area of the amorphous halo (A_{am}) from the area of the whole diffraction profiles $A_c = A_t - A_{am}$. The scattering of the amorphous phase was determined for each sample by recording the WAXS profile at a temperature higher than its melting temperature.

Tensile tests were performed on melt crystallized compression-molded films obtained by slow cooling from the melt, soon after their preparation, with a universal tensile test machine Zwick/Roell BTC-FR2.5TH.D09, following the standard test method for tensile properties of thin plastic sheeting ASTM D882. Rectangular specimens 100 mm long and 5 mm wide, cut from the compression-molded films using a die-cutter, were stretched up to the break while recording the stress-strain curves to determine tensile test parameters, such as the stress and strain at break (σ_b and ε_b) and at the yield point (σ_y and ε_y) and the tension set after breaking (t_b). The values of Young's modulus (E) were determined in independent experiments.

The strain at break ε_b was evaluated as $\varepsilon_b=((L_f-L_0)/L_0) \times 100$ where L_0 and L_f are the initial and final lengths of the specimen, respectively. The tension set after break t_b, which represents a measurement of the elastomeric properties of the samples, were determined following the procedure described in the ASTM D412. Ten minutes after breaking, the two pieces of the broken specimen were fit carefully together so that they are in contact over the full area of the break and the final total length L_r was measured. The tension set after breaking was calculated as $t_b=((L_r-L_0)/L_0) \times 100$. In the tensile tests, the ratio between the drawing rate and the initial length was fixed equal to 10 mm/(mm·min) during recording the stress-strain curves and equal to 0.1 mm/(mm·min) in the evaluation of Young's moduli. The reported stress-strain curves and the values of mechanical properties are averaged over at least five independent experiments.

ATR-IR spectra of polymers were acquired on a Perkin Elmer Spectrum 100 instrument. Quantitative IR analysis allowed calculation of the CO incorporation as described in detail in previously.[3] For calculation of the carbon monoxide incorporation, the ratio of the integral of the C=O signal (integration area: band between 1692-1715 cm^{-1} ± 65 cm^{-1}) to the integral of the C-H signal of the PE at ~2915 cm^{-1} (integration area: 2740-3030 cm^{-1}) was calculated and referenced with linear polyketone samples with known C=O content synthesized via ADMET copolymerization and subsequent hydrogenation.[4]

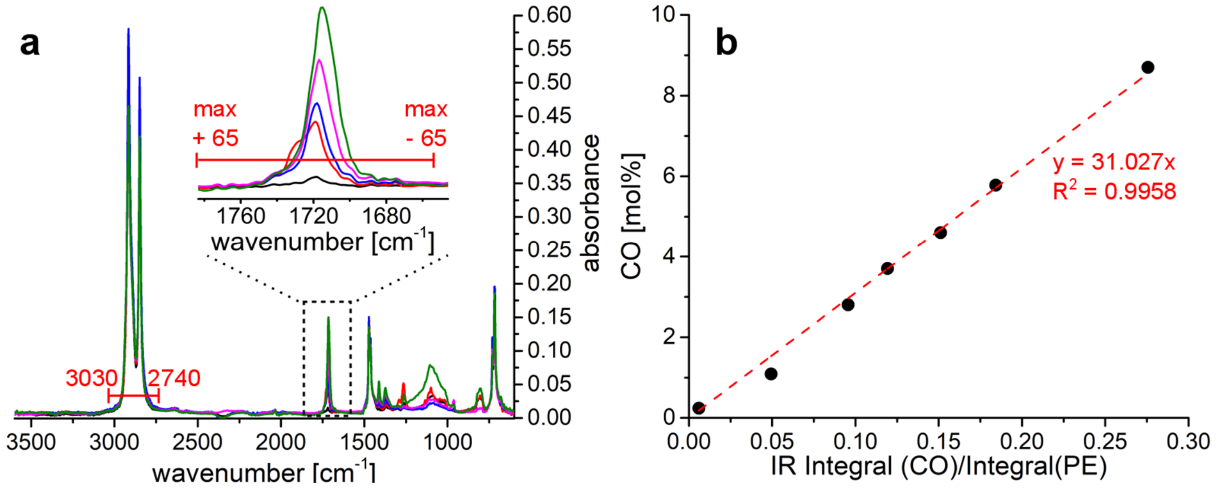

Figure S1. Referencing of IR spectra. **a:** Polyketones with known carbonyl contents were analyzed by ATR-IR. The samples were synthesized via ADMET copolymerization of docosa-1,21-dien-11-one and undeca-1,10-diene followed by hydrogenation.[4] The range of integration for the determination of signal intensity ratios for carbonyl stretching vibrations (spectral position with max. absorbance ± 65 cm^{-1}) vs. the polyethylene C-H vibration (2740-3030 cm^{-1}) are depicted in red. **b:** The integral intensity ratio is directly proportional to the concentration of C=O groups in the polymer χ. That is, $\chi \approx n_{CO}/n_{C_2H_4}$ which is a valid approximation if $n_{C_2H_4} >> n_{CO}$. Figure reprinted with permission from *Science* 2021, 374, 604 – 607. Copyright © 2021, The American Association for the Advancement of Science).[3]

1.2 General polymerization procedure

All polymerization experiments were carried out in a high-pressure polymerization set-up consisting of a 300 mL MiniClave by BüchiGlasUster equipped with a Cyclone 075 magnetically coupled mechanical pitched blade stirrer, in- and outlet valves, a temperature sensor, a digital pressure sensor, a Julabo CF41 thermostat and continuous (co-)monomer gas feeds. Gas flows of ethylene and carbon monoxide were individually monitored and regulated by El Flow mass flow controllers by Bronkhorst. Gas feeds and temperature were controlled *via* LabVision software automatization by Hitec Zang.

The reactor was evacuated and purged with nitrogen three times prior to the reaction. 100 mL of dried and deoxygenated toluene were transferred into the reactor vessel by cannula and heated to the desired reaction temperature, before the precatalyst, dissolved in 4 mL of toluene and the respective amount of norbornene dissolved in toluene (conc. ~ 0.1 g/mL) were injected into the reactor by syringe, consecutively. The stirring rate was adjusted to 1000 rpm and the reactor was pressurized with the desired mixture of carbon monoxide and ethylene. This mixture was further fed to the reactor by the automated mass flow controllers to maintain a stable pressure (thus replenishing consumed gaseous monomer) over the course of the entire reaction. After the desired polymerization time, the reactor was vented and cooled. The obtained polymer was precipitated in methanol, filtered off, washed thoroughly with methanol and vacuum dried at 60 °C for > 24 h. Reference copolymerizations of ethylene-norbornene and ethylene-CO were carried out following the same protocol.

2. Polymer characterization

2.1 Supplementary IR spectra of polymers

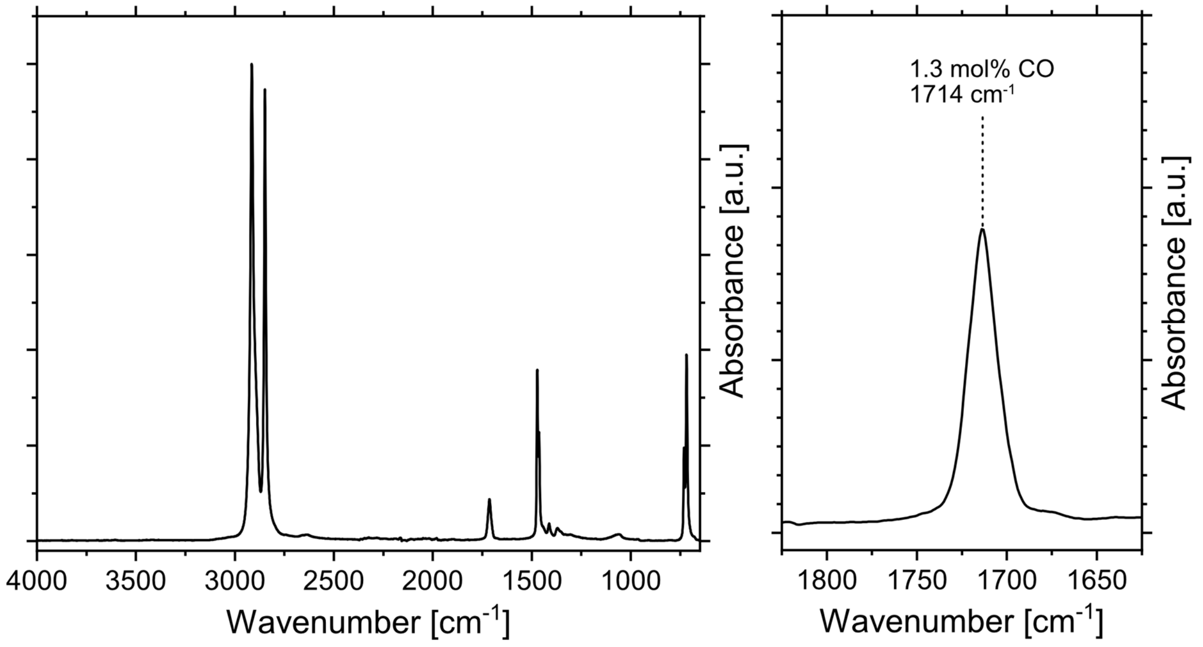

Figure S2. ATR-IR spectrum (left) and detailed carbonyl region (right) of a reference keto-PE with 1.3 mol% CO incorporation as obtained from copolymerization with precatalyst **1** (Table 1, entry 2).

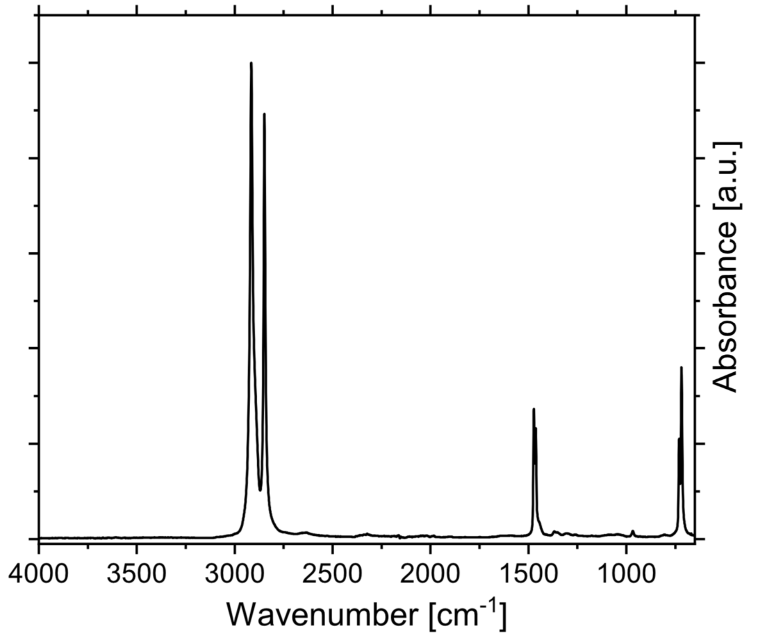

Figure S3. ATR-IR spectrum of a reference ethylene-norbornene copolymer with 0.7 mol% norbornene incorporation as obtained from copolymerization with precatalyst **1** (Table 1, entry 1).

2.2 Supplementary NMR spectra of polymers

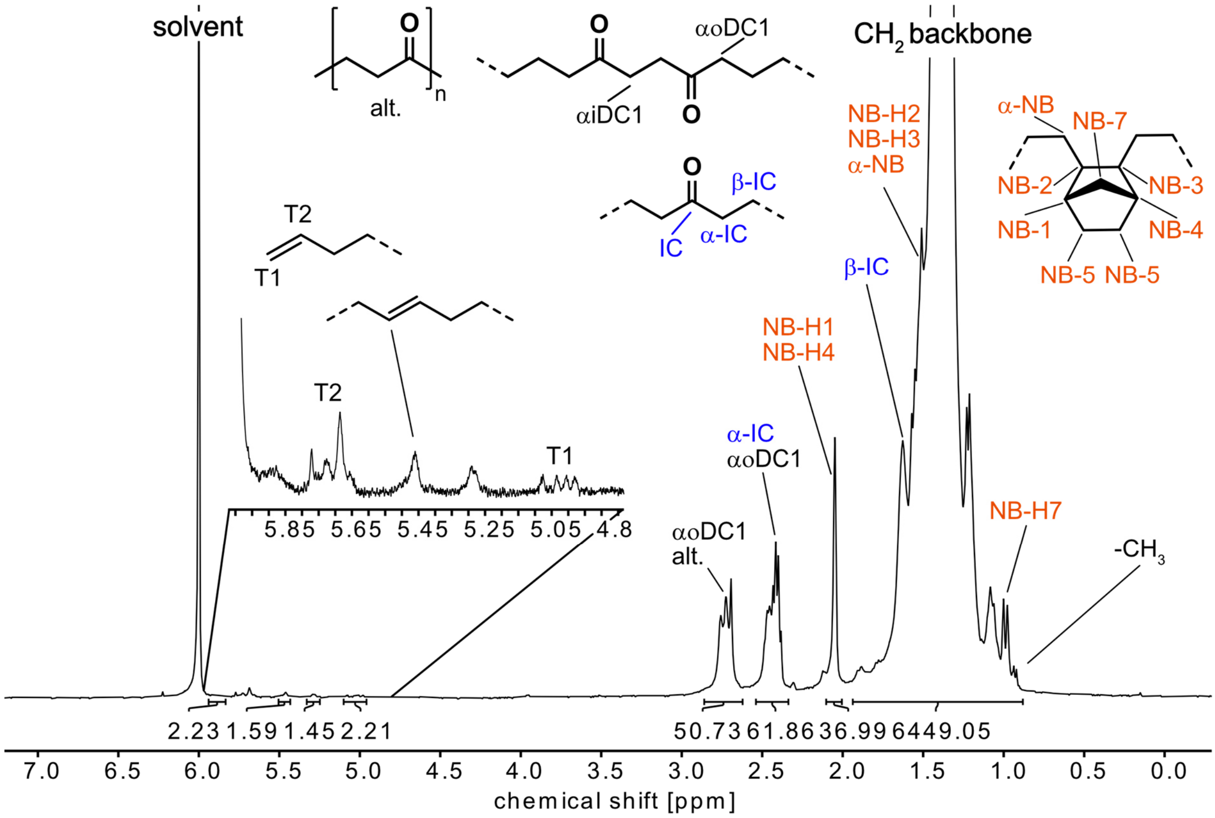

Figure S4. ^{1}H NMR spectrum (400 MHz, 383 K, $C_2D_2Cl_4$) of a ^{13}CO labelled ethylene-norbornene-CO terpolymer with 1.0 mol% C=O and 1.2 mol% norbornene incorporation obtained from polymerization with complex **1** (Table 1, entry 6).

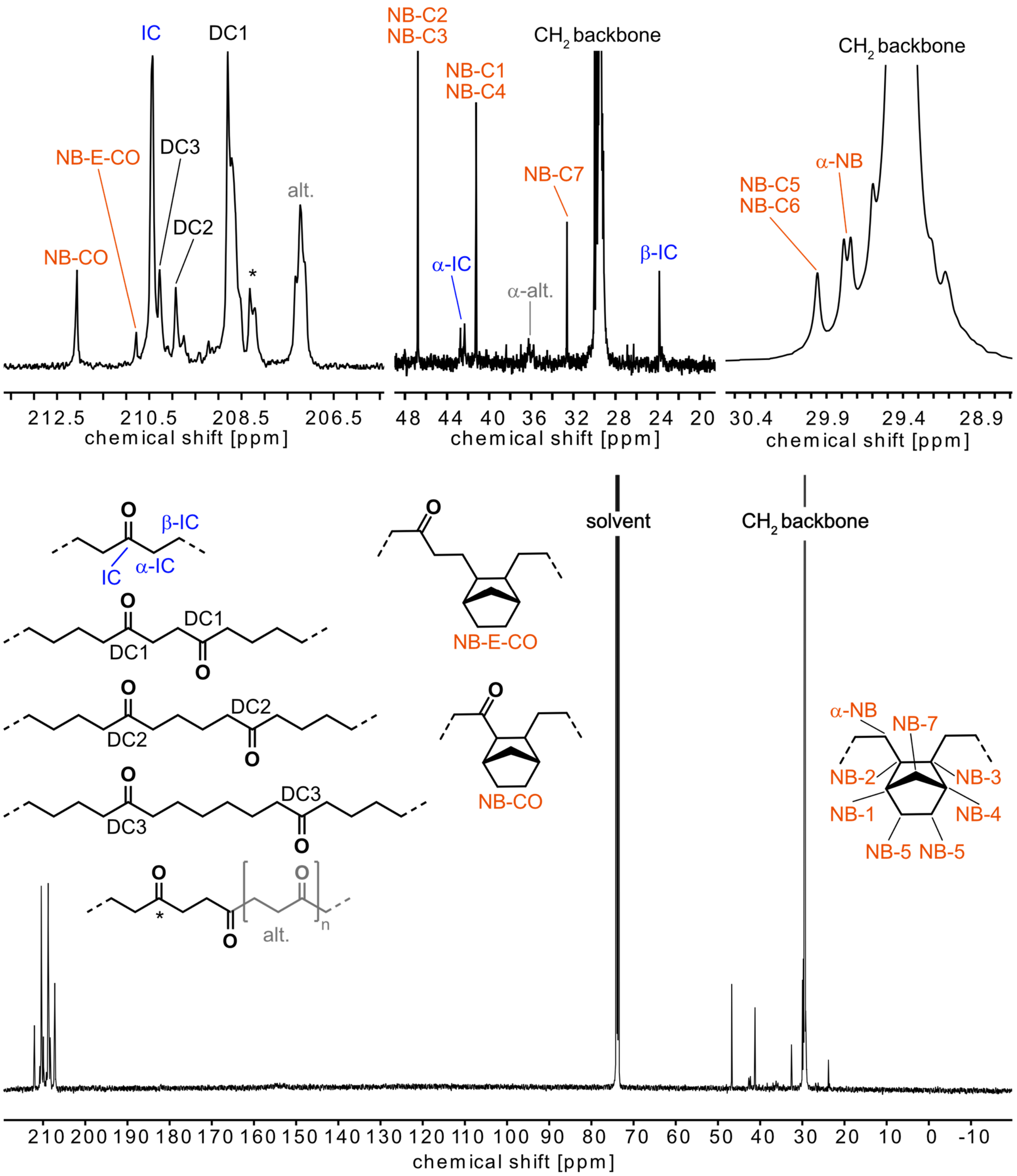

Figure S5. Full ^{13}C {^{1}H} NMR spectrum (101 MHz, 383 K, $C_2D_2Cl_4$) with enlargements of a ^{13}CO labelled ethylene-norbornene-CO terpolymer with 1.0 mol% C=O and 1.2 mol% norbornene incorporation obtained from polymerization with complex **1** (*cf.* Table 1, entry 6).

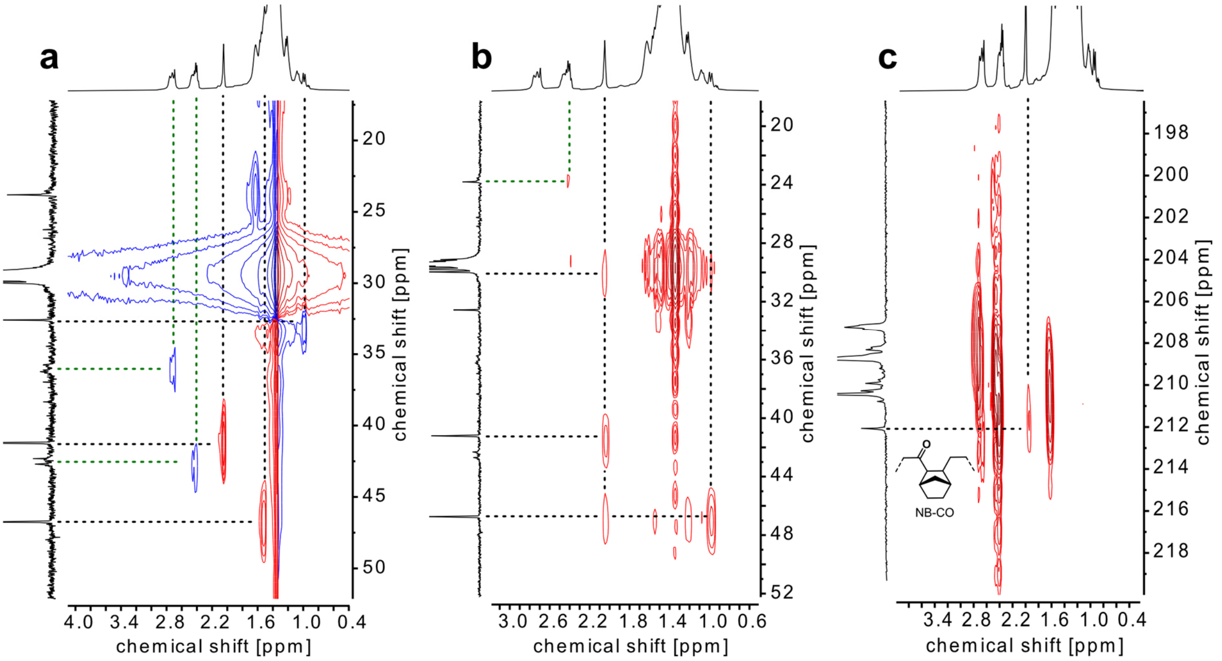

Figure S6. 2D NMR spectra (383 K, $C_2D_2Cl_4$) of a ^{13}CO labelled ethylene-norbornene-CO terpolymer (*cf.* Table 1, entry 6) with 1.0 mol% C=O and 1.2 mol% norbornene incorporation. Black lines indicate correlations of norbornene signals, green lines indicate correlations of keto-units. For full ^{1}H and ^{13}C assignment see **Figure S4** and **Figure S5**) a: ^{1}H-^{13}C HSQC, alkyl region (18 – 52 ppm, 0.4 – 4 ppm). b: ^{1}H-^{13}C HMBC, alkyl region (18 – 52 ppm, 0.5 – 3.2 ppm). ^{1}H-^{13}C HMBC of carbonyls (196 – 220 ppm, 0.2 – 4.0 ppm).

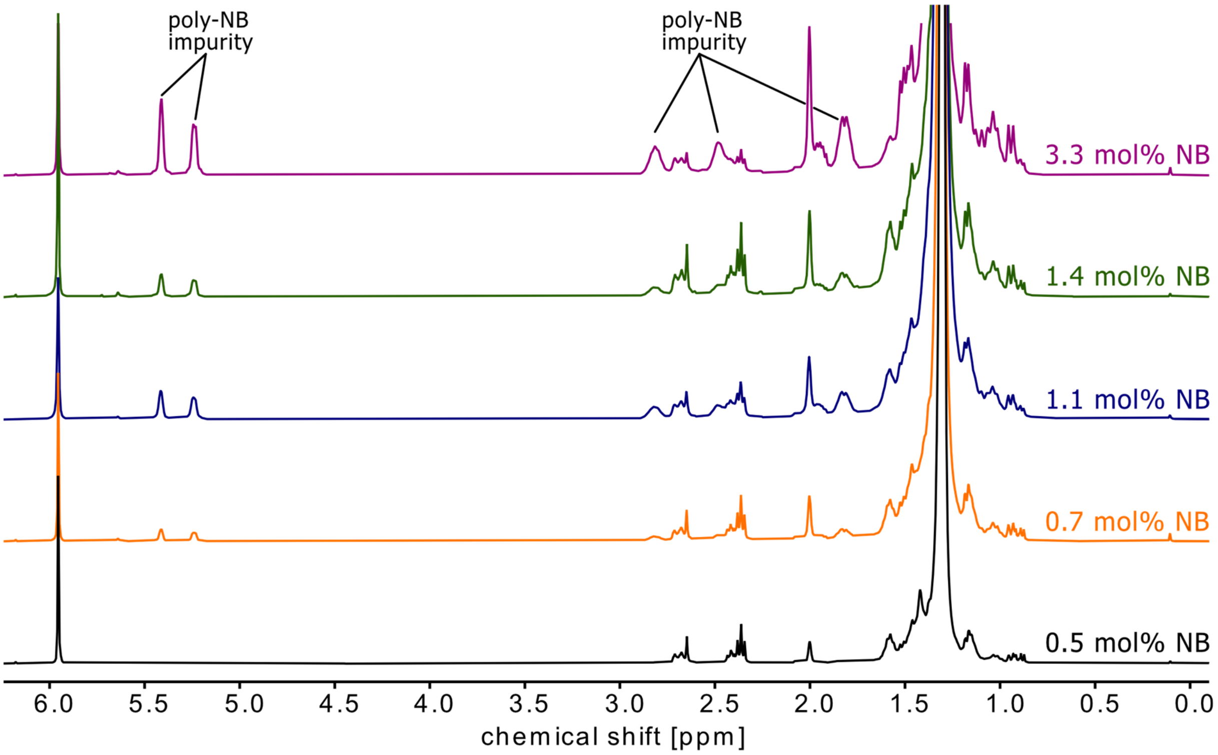

Figure S7. Stacked ^{1}H NMR spectra (400 MHz, 383 K, $C_2D_2Cl_4$) of ethylene-norbornene-CO terpolymers with different norbornene incorporations, as determined from ^{1}H NMR spectroscopy. Note the observation of variable amounts of poly-norbornene impurities[5] which can be removed by washing with toluene (*vide infra* for discussion about poly-NB removal).

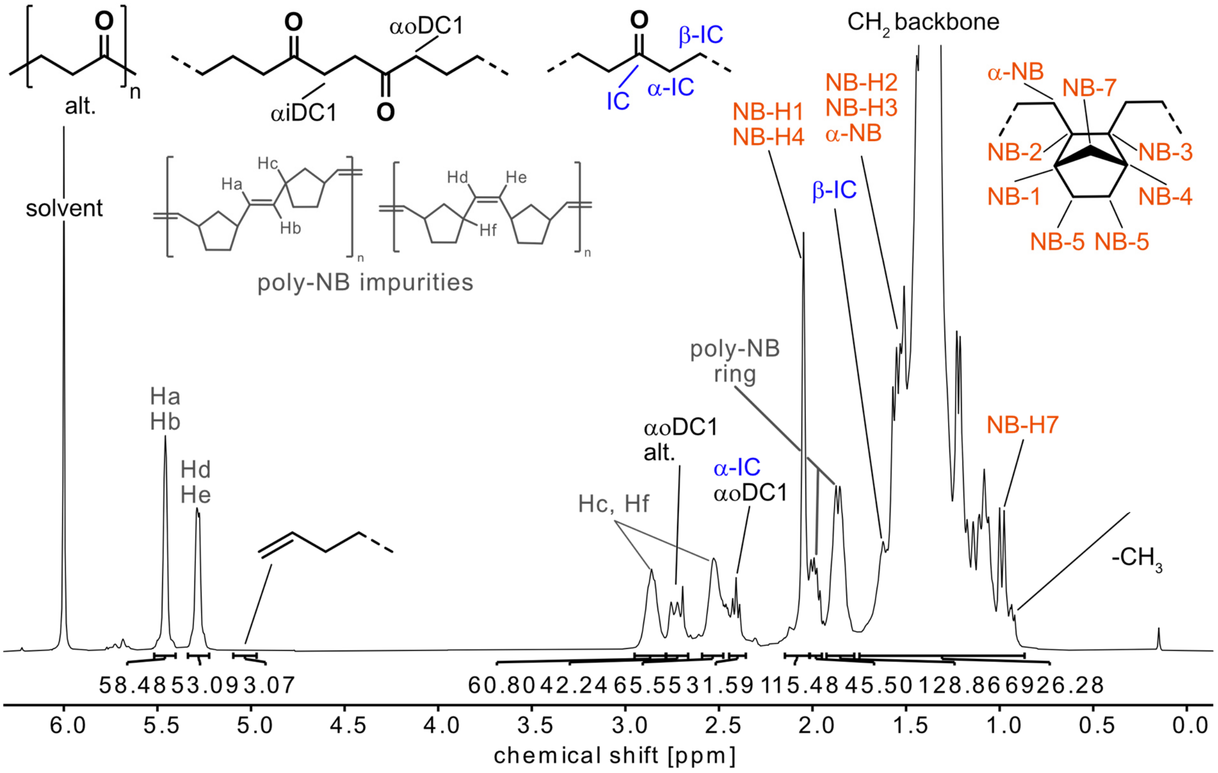

Figure S8. ^1H NMR spectrum (400 MHz, 383 K, $C_2D_2Cl_4$) of an ethylene-norbornene-CO terpolymer (*cf.* Table 1, entry 9) with 1.1 mol% C=O and 3.3 mol% norbornene incorporation. Note the observation of poly-norbornene impurity (1.4 mol%, highest observed ratio).[5] *Vide infra* for discussion about removal.

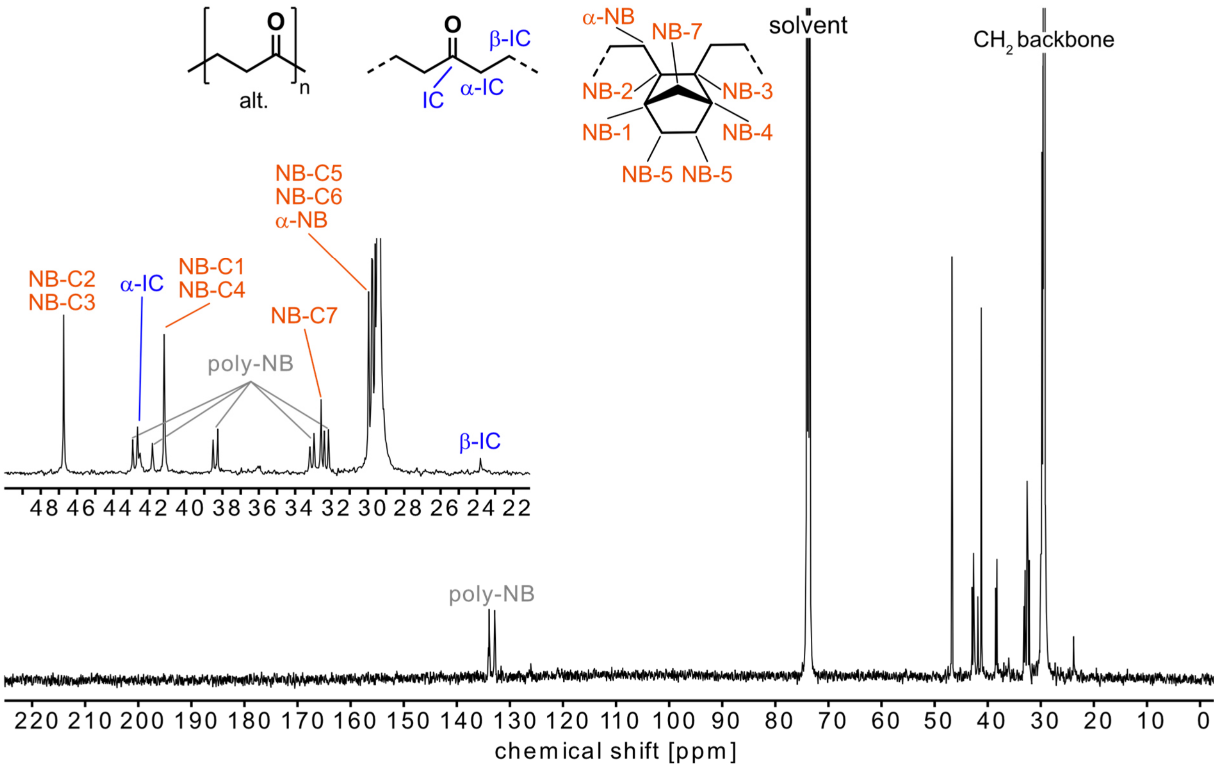

Figure S9. ^{13}C NMR spectrum (101 MHz, 383 K, $C_2D_2Cl_4$) of an ethylene-norbornene-CO terpolymer (*cf.* Table 1, entry 9) with 1.1 mol% C=O and 3.3 mol% norbornene incorporation. Note the observation of poly-norbornene impurity (1.4 mol%, highest observed ratio).[5] *Vide infra* for discussion about removal.

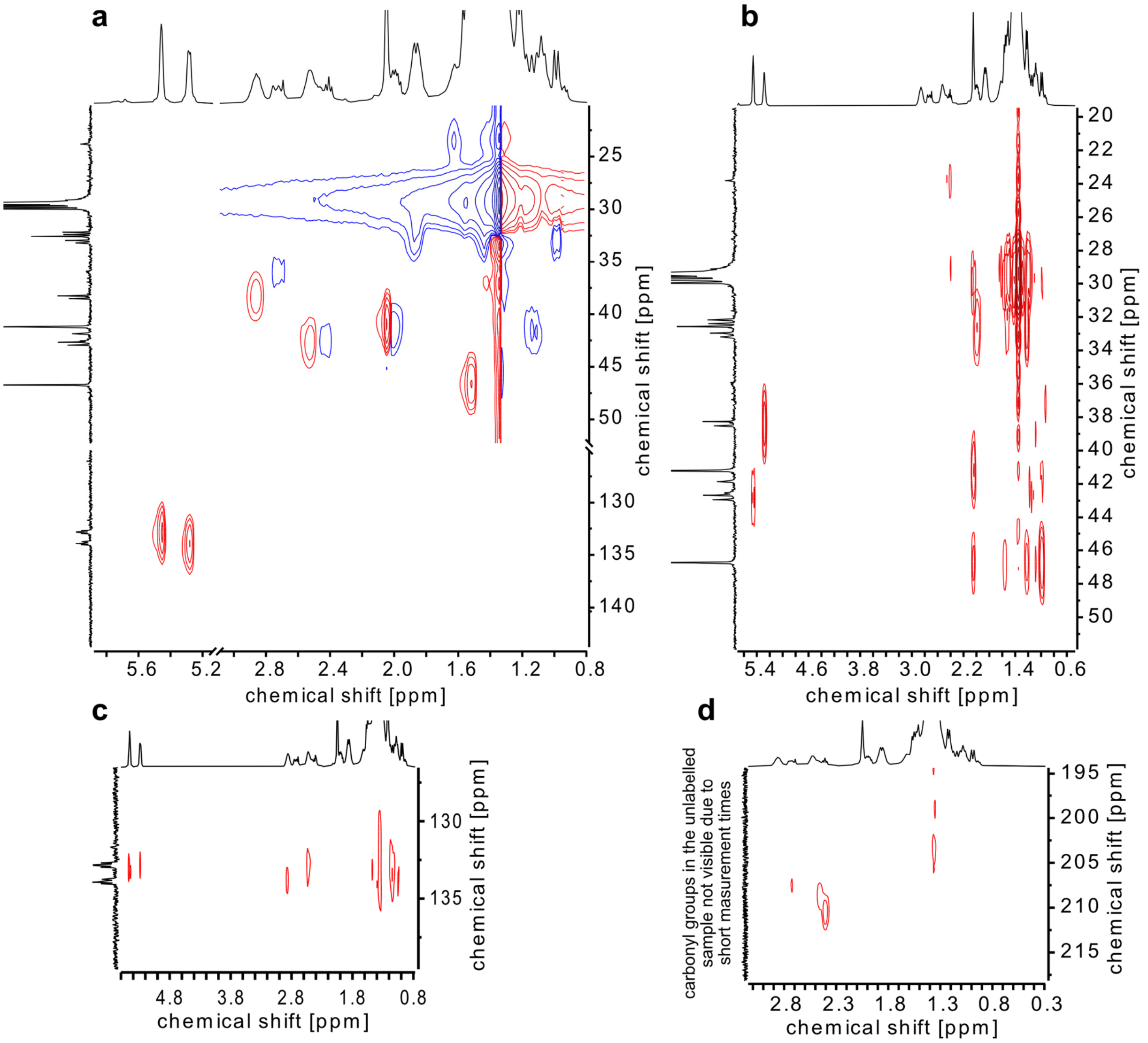

Figure S10. 2D NMR spectra (383 K, $C_2D_2Cl_4$) of an ethylene-norbornene-CO terpolymer (*cf.* Table 1 entry 9) with 1.1 mol% C=O and 3.3 mol% norbornene incorporation showing the expected correlations as observed for the ^{13}C labelled terpolymer (*cf.* **Figure S6**) but no correlations between terpolymer resonances and poly-norbornene impurities. For full ^{1}H and ^{13}C assignment see **Figure S4** and **Figure S5**) **a:** ^{1}H-^{13}C HSQC (18 – 52 ppm and 120 – 145 ppm, 0.8 – 3.0 ppm and 5.2 – 5.8 ppm). **b:** ^{1}H-^{13}C HMBC, alkyl region (19 – 52 ppm, 0.5 – 5.8 ppm). **c:** ^{1}H-^{13}C HMBC of poly-NB olefins (127 – 140 ppm, 0.8 – 5.6 ppm). **d:** ^{1}H-^{13}C HMBC of carbonyl region (196 – 220 ppm, 0.3 – 3.0 ppm), note that observation of carbonyl groups in the non-labelled polymer would require much longer measurement times.

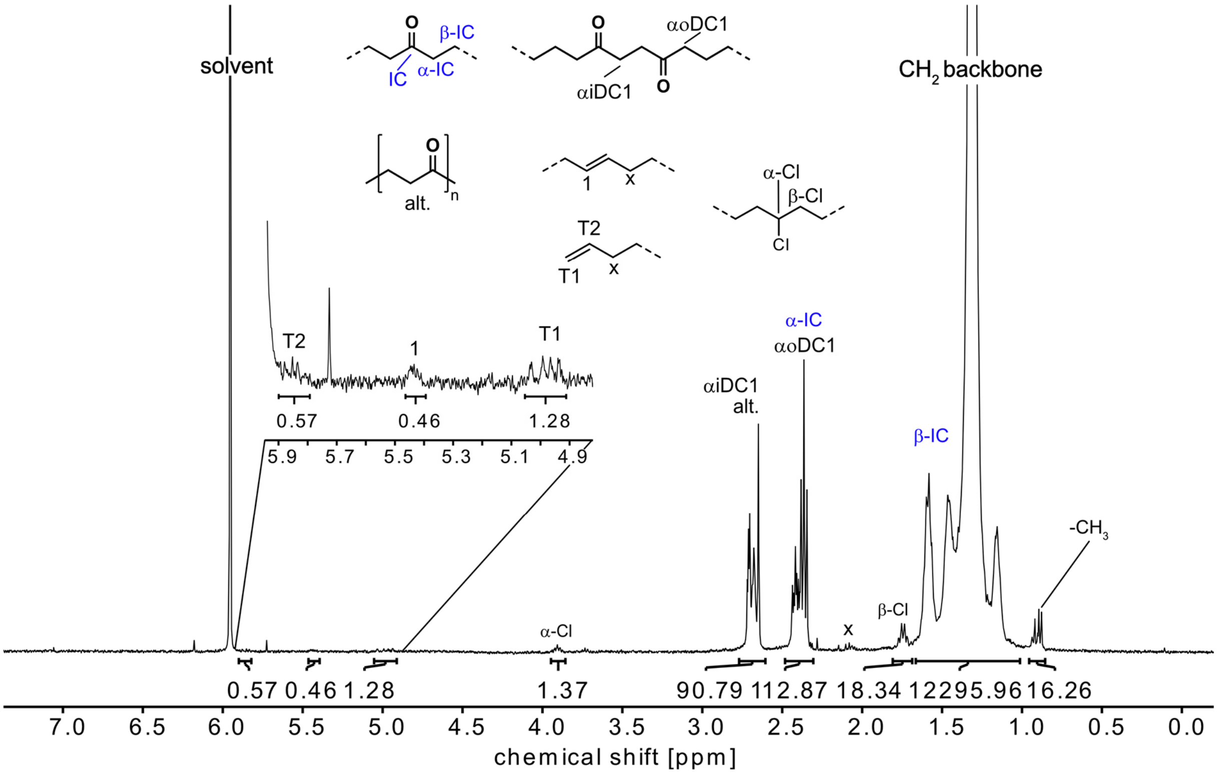

Figure S11.^{1}H NMR spectrum (400 MHz, 383 K, $C_2D_2Cl_4$) of a reference keto-PE obtained from ethylene-CO copolymerization with complex **1** (*cf.* Table 1, entry 2). Note the occurrence of backbone chlorination[6] by the solvent due to the long high-temperature measurement required for detection of endgroups.

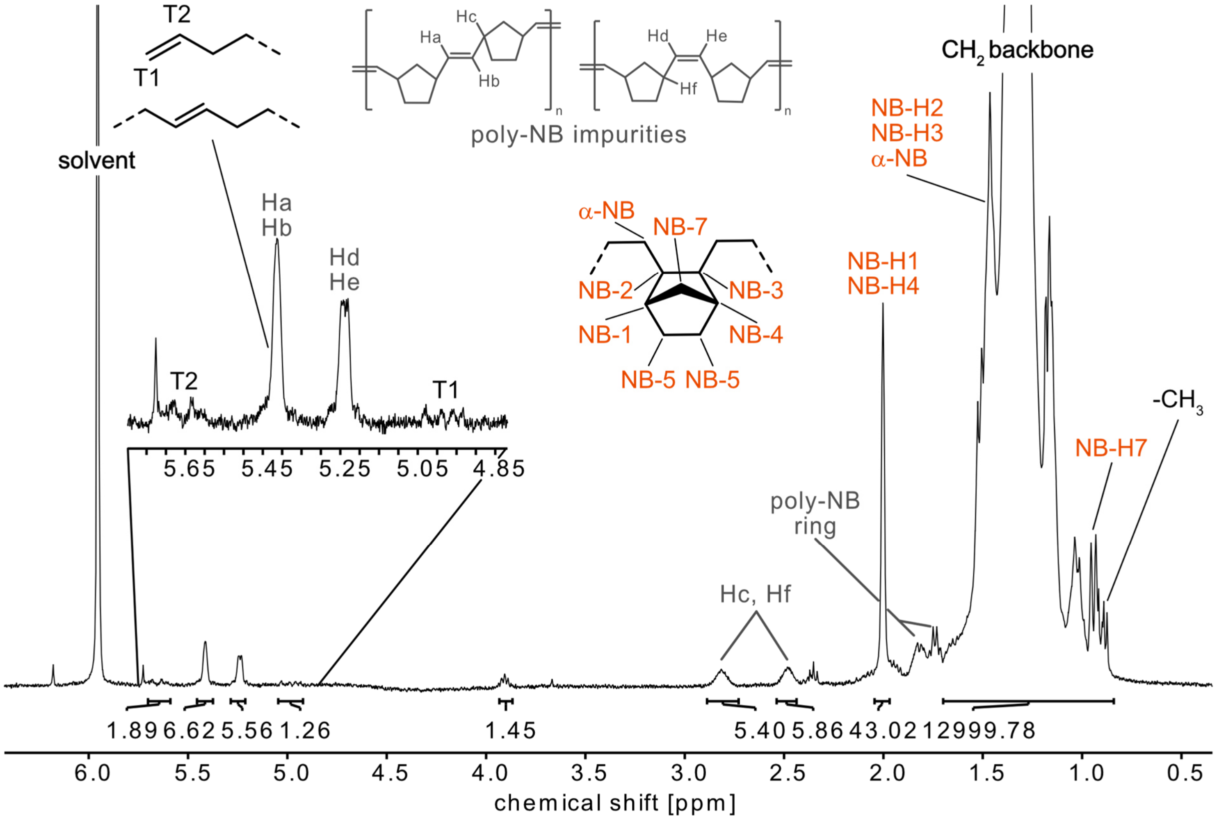

Figure S12.^{1}H NMR spectrum (400 MHz, 383 K, $C_2D_2Cl_4$) of a reference ethylene-norbornene copolymer with 0.7 mol% NB incorporation obtained from copolymerization with complex **1** (*cf.* Table 1, entry 1). Note the observation of poly-norbornene impurity (0.1 mol%).[5] *Vide infra* for discussion about removal.

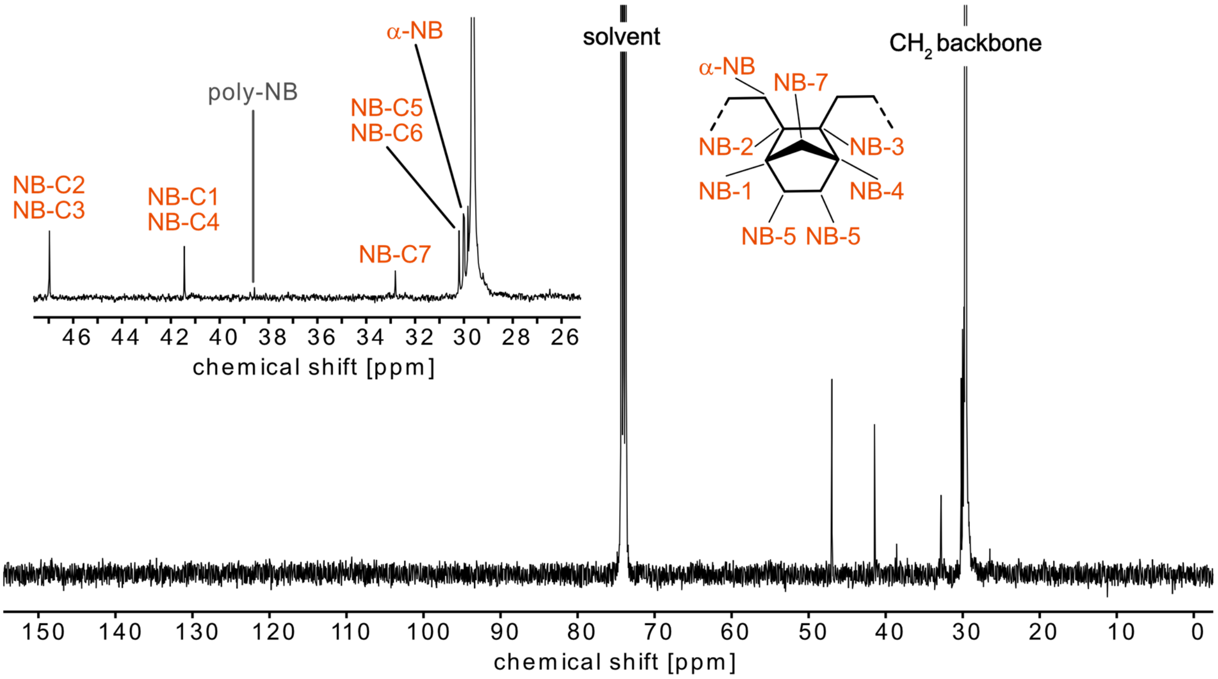

Figure S13.^{13}C NMR spectrum (101 MHz, 383 K, $C_2D_2Cl_4$) of a reference ethylene-norbornene copolymer with 0.7 mol% NB incorporation obtained from copolymerization with complex **1** (*cf.* Table 1, entry 1). Note the observation of poly-norbornene impurity (0.1 mol%).[5] *Vide infra* for discussion about removal.

2.3 SEC traces of polymers

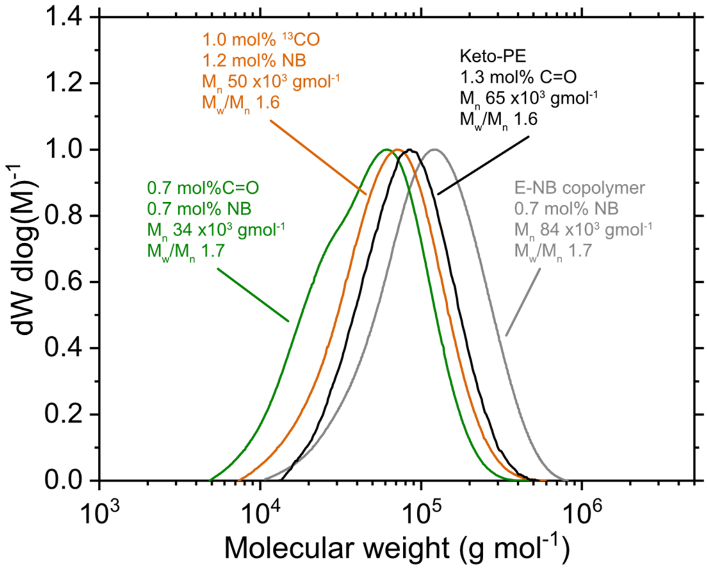

Figure S14. SEC traces of two crystallinity reduced keto-PEs (Table 1 entries 4 and 6), a reference ethylene-NB copolymer (Table 1, entry1) and a reference keto-PE (Table 1, entry 2).

2.4 Thermal properties from differential scanning calorimetry (DSC)

Table S1. Thermal properties and degrees of crystallinity (x^{DSC}) of samples reported in Table 1.

Entry No.	X (CO)‡ [mol%]	X (NB)# [mol%]	T_c [°C]	ΔH_C [J/g]	T_m [°C]	ΔH_m [J/g]	x_c^{DSC} [mol%]**
1	-	0.7	106	125	124	122	41
2	1.3	-	109	186	136	186	63
3	0.9 (1.1)	0.5	109	154	126	152	51
4	0.7 (1.1)	0.7	108	142	126	141	47
5	0.7 (0.7)	1.1	105	132	124	128	43
6§	1.0 (1.7)	1.2 (1.1)	98	126	122	123	43
7	0.9 (1.4)	1.4	104	132	124	128	43
8	0.7 (1.3)	1.3	99	118	122	104	35
9	1.1 (1.0)	3.3	85/106	90	97/124	89	30

‡ determined by ATR-IR spectroscopy. In brackets: Incorporation determined by ^{1}H NMR spectroscopy by integration of the ^{1}H signals of α-carbonyl CH_2 (CO) in relation to the overall integral. # determined by ^{1}H NMR spectroscopy by integration of ^{1}H signals of norbornene H1&4 protons at 2.00 ppm. In brackets: Incorporation determined by quantitative ^{13}C NMR spectroscopy. *Determined as described in section 1.1.2. § ^{13}CO employed as comonomer.

2.5 WAXS data of polymers and crystallinity evaluation

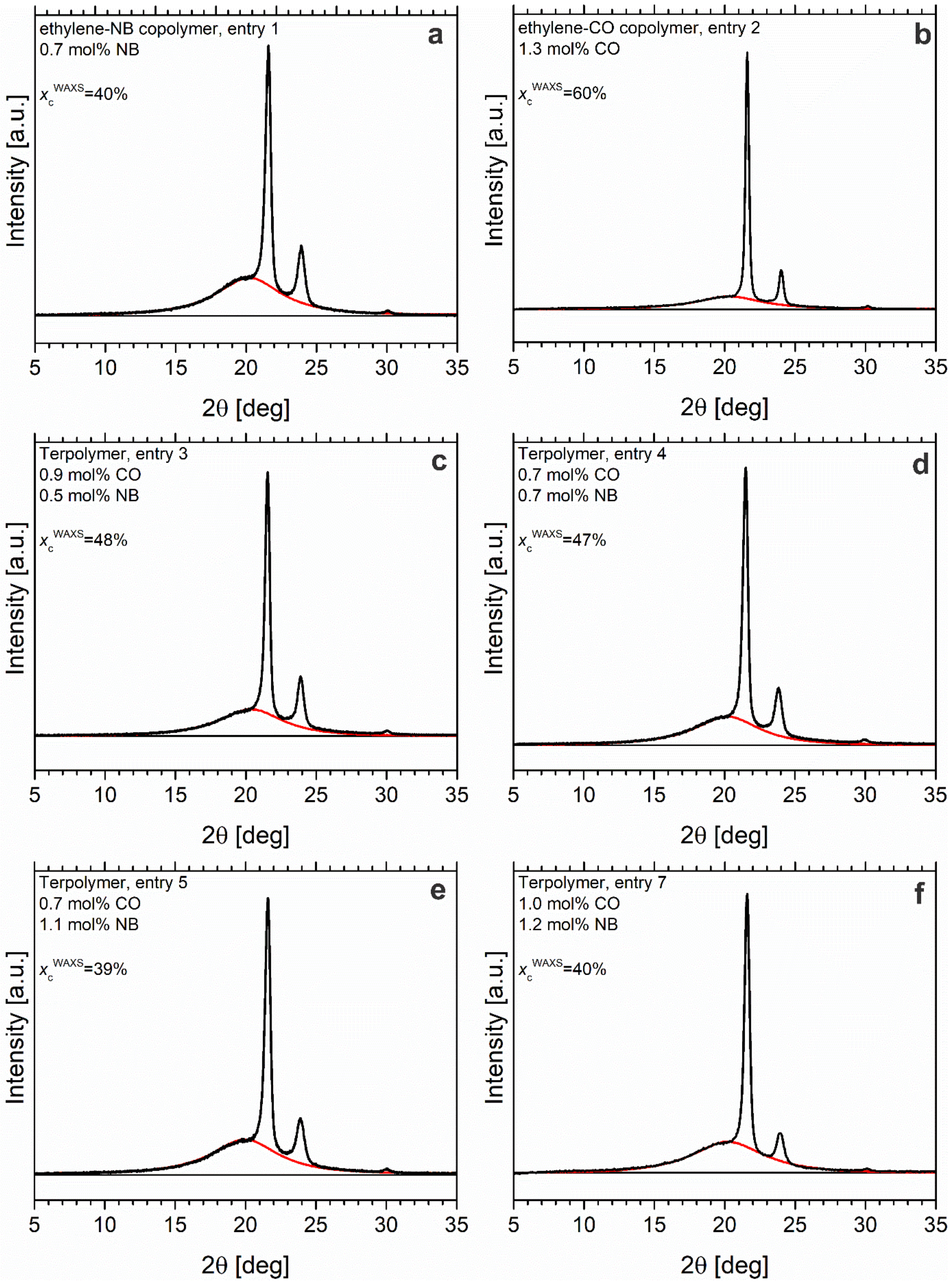

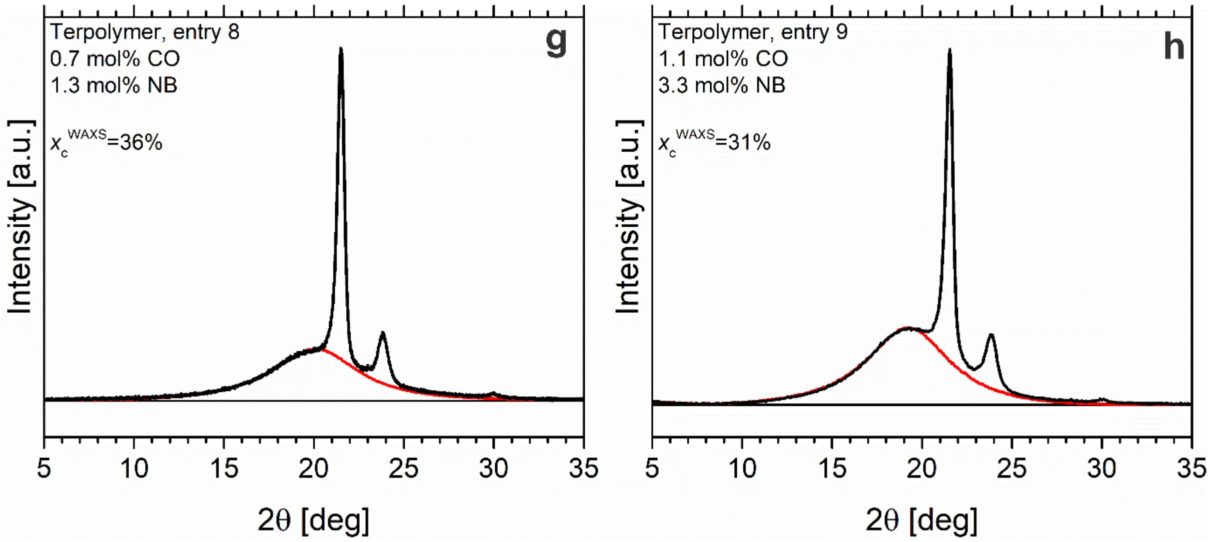

Figure S15. Evaluation of the degrees of crystallinity (x_c^{WAXS}) from WAXS profiles of melt-crystallized samples 1 (a), 2 (b), 3 (c), 4 (d), 5 (e), 7 (f), 8 (g) and 9 (h) with the indicated CO and NB concentrations (Sample number indicates entry number in Table 1). For each sample, the scattering profile of the amorphous phase scaled under the diffraction profiles of the semicrystalline samples for the determination of the degree of crystallinity, as described in Section 1.1.2, are indicated as red lines.

2.6 Tensile properties of crystallinity reduced keto-PEs

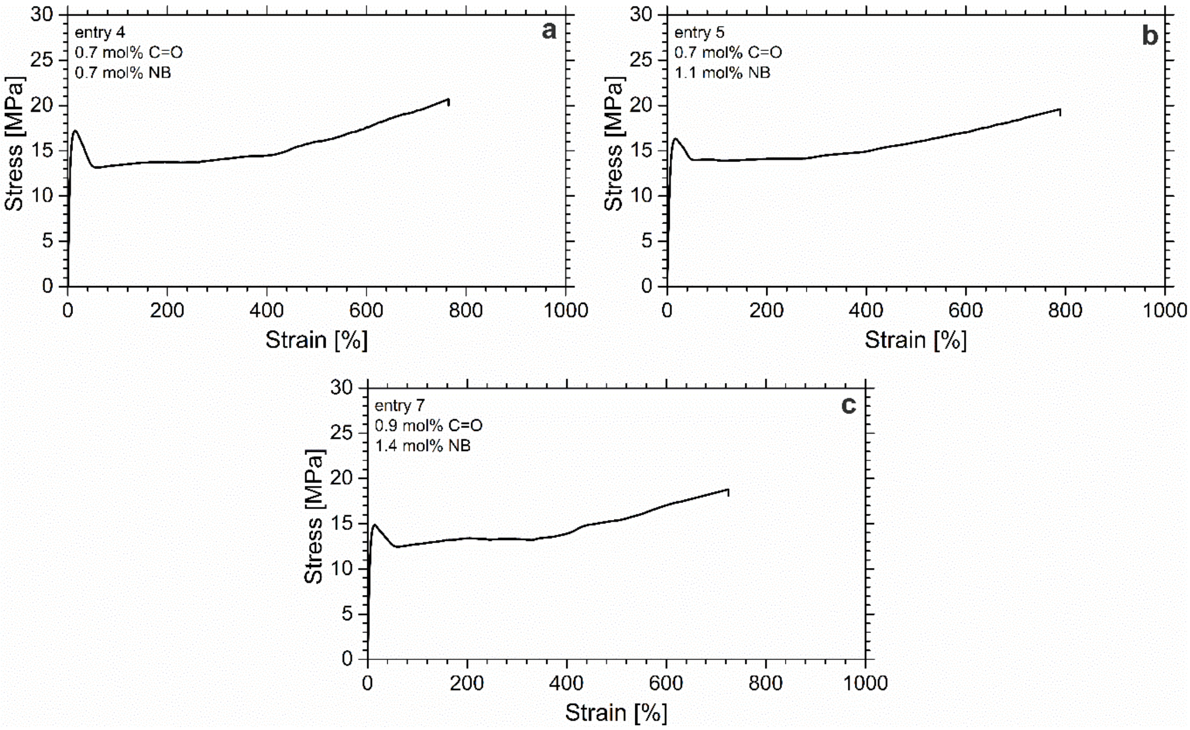

Figure S16. Stress-strain curves of samples 4 (a), 5 (b) and 7 (c) with the indicated CO and NB concentrations.

Table S2. Tensile test results extracted from stress-strain curves of Figures 5 and S16.

Entry No.	X (CO)‡ [mol%]	X (NB)# [mol%]	E [MPa]	ε_y [%]	σ_y [MPa]	ε_b [%]	σ_b [MPa]	t_b [%]
1	-	0.7	345 ± 30	17 ± 2	16 ± 3	640 ± 50	25 ± 5	320 ± 50
2	1.3	-	610 ± 25	12 ± 1	23 ± 1	710 ± 90	25 ± 2	510 ± 25
3	0.9 (1.1)	0.5	325 ± 12	13 ± 1	18 ± 1	950 ± 30	22 ± 1	460 ± 40
4	0.7 (1.1)	0.7	324 ± 3	15 ± 1	17 ± 1	765 ± 20	21 ± 2	390 ± 20
5	0.7 (0.7)	1.1	265 ± 20	17 ± 1	16 ± 1	790 ± 50	20 ± 2	370 ± 10
7	0.9 (1.4)	1.4	250 ± 15	17 ± 1	15 ± 1	725 ± 60	19 ± 1	360 ± 50
8	0.7 (1.3)	1.3	215 ± 6	18 ± 1	13 ± 1	670 ± 50	17 ± 1	260 ± 30
9	1.1 (1.0)	3.3	200 ± 9	10 ± 1	10 ± 1	760 ± 30	25 ± 4	230 ± 10
HDPE	-	-	745 ± 20	10 ± 1	25 ± 1	670 ± 10	21 ± 2	560 ± 30
LDPE	-	-	170 ± 15	45 ± 4	11.5 ± 1	950 ± 20	20 ± 4	410± 20

‡ determined by ATR-IR spectroscopy. In brackets: Incorporation determined by ^{1}H NMR spectroscopy by integration of the ^{1}H signals of α-carbonyl CH_2 (CO) in relation to the overall integral. # determined by ^{1}H NMR spectroscopy by integration of ^{1}H signals of norbornene H1&4 protons at 2.00 ppm. In brackets: Incorporation determined by quantitative ^{13}C NMR spectroscopy.

2.7 Removal of polynorbornene impurities

Evaluation of ^{1}H NMR spectra showed the occurrence of poly-norbornene formation in most polymer samples (*cf.* **Figure S7**), likely due to side reactions or impurities at the employed high polymerization temperatures.[5] Nevertheless, fractions of formed poly-norbornene fractions are small in comparison to the overall yield (0.1 – max. 1.2 mol%, based on ^{1}H NMR spectroscopy) and do not correlate with the initially applied norbornene concentration in the reaction mixture. Due to the completely amorphous nature, the poly-norbornene fractions could be removed by washing the obtained polymers with toluene at room temperature. To exclude effects of the initially contained polynorbornene like artificially reduced crystallinity due to an additional amorphous fraction, evaluation of the thermal, mechanical and solid-state properties of the terpolymers before and after washing were performed. These showed the same materials properties before and after washing, evidencing no adverse effect of the small amount of initially contained polynorbornene.

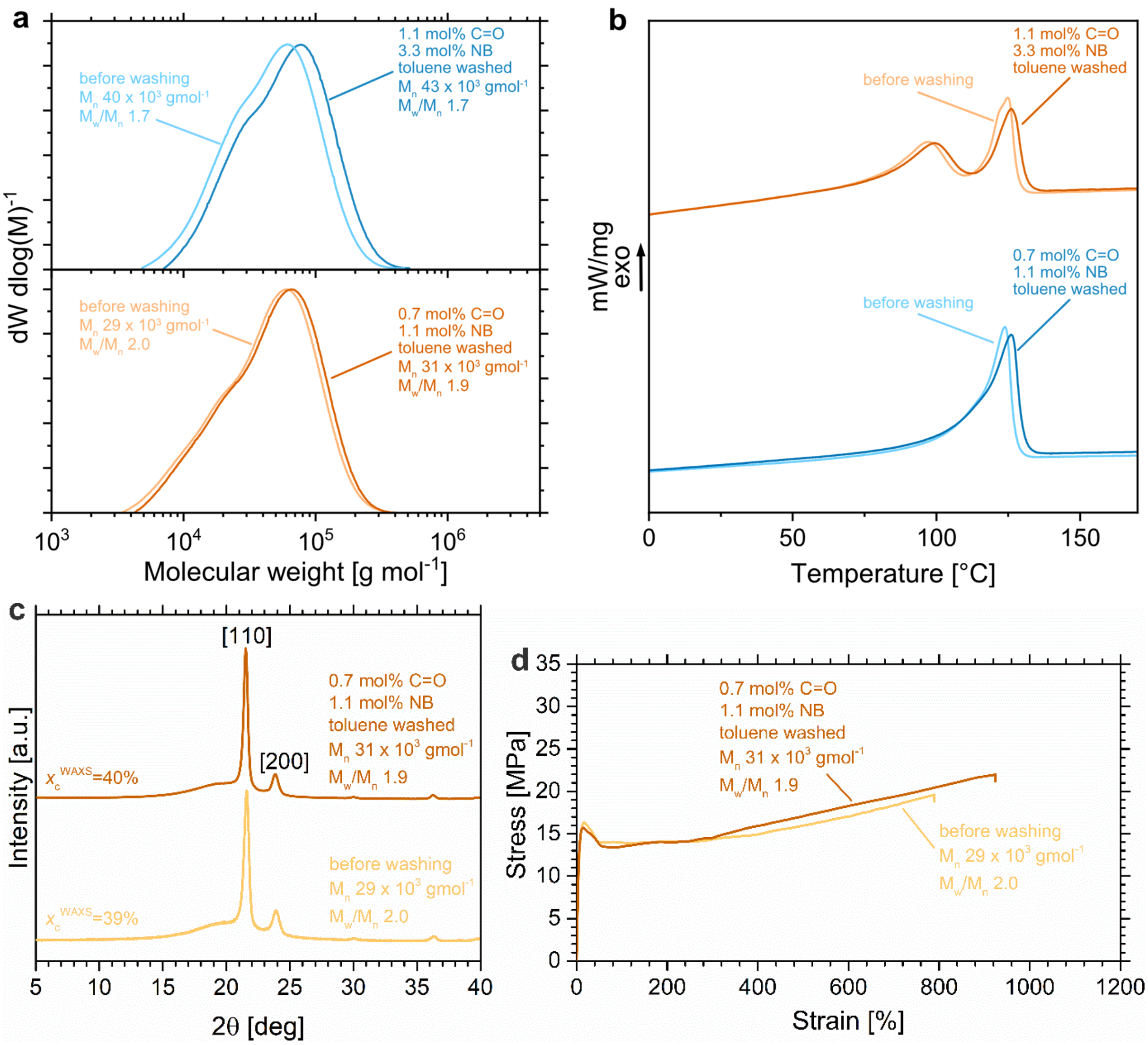

Figure S17. Analysis of terpolymers before and after repeated washing with toluene to remove amorphous polynorbornene impurities. **a:** SEC traces, shifted vertically for clarity. **b:** DSC traces, shifted vertically for clarity. **c:** WAXS profiles of melt-crystallized samples, shifted vertically for clarity. **d:** stress-strain curves of compression-molded films.

Table S3. Tensile test results extracted from stress-strain curves of Figure S17, d.

#	X (CO)‡ [mol%]	X (NB)# [mol%]	*E* [MPa]	ε_y [%]	σ_y [MPa]	ε_b [%]	σ_b [MPa]	t_b [%]
5	0.7 (0.7)	1.1	265 ± 20	17 ± 1	16 ± 1	790 ± 50	20 ± 2	370 ± 10
5*	0.7 (0.7)	1.1	285 ± 20	17 ± 1	16 ± 1	925 ± 70	22 ± 2	380 ± 15

‡ determined by ATR-IR spectroscopy. In brackets: Incorporation determined by ^{1}H NMR spectroscopy by integration of the ^{1}H signals of α-carbonyl CH_2 (CO) in relation to the overall integral. # determined by ^{1}H NMR spectroscopy by integration of ^{1}H signals of norbornene H1&4 protons at 2.00 ppm. In brackets: Incorporation determined by quantitative ^{13}C NMR spectroscopy. *sample tested after washing with toluene.

3. Supporting References

[1] Zhang, Y.; Mu, H.; Wang, X.; Pan, L.; Li, Y. Elaborate Tuning in Ligand Makes a Big Difference in Catalytic Performance: Bulky Nickel Catalysts for (Co)polymerization of Ethylene with Promising Vinyl Polar Monomers. *ChemCatChem* **2019**, *11* (9), 2329–2340. DOI: 10.1002/cctc.201900265.

[2] Quirk, R. P.; Alsamarrie, M. A. A. Physical constants of polyethylene. In *Polymer Handbook,* 3. ed.; Bandrup, J., Immergut, E. H., Eds.; Wiley, 1989.

[3] Baur, M.; Lin, F.; Morgen, T. O.; Odenwald, L.; Mecking, S. Polyethylene materials with in-chain ketones from nonalternating catalytic copolymerization. *Science* **2021**, *374* (6567), 604–607. DOI: 10.1126/science.abi8183.

[4] Ortmann, P.; Wimmer, F. P.; Mecking, S. Long-Spaced Polyketones from ADMET Copolymerizations as Ideal Models for Ethylene/CO Copolymers. *ACS Macro Lett.* **2015**, *4* (7), 704–707. DOI: 10.1021/acsmacrolett.5b00324.

[5] Sacchi, M. C.; Sonzogni, M.; Losio, S.; Forlini, F.; Locatelli, P.; Tritto, I.; Licchelli, M. Vinylic Polymerization of Norbornene by Late Transition Metal-Based Catalysis. *Macromol. Chem. Phys.* **2001**, *202* (10), 2052–2058. DOI: 10.1002/1521-3935(20010601)202:10<2052:AID-MACP2052>3.0.CO;2-P.

[6] Brandolini, A. J.; Hills, D. D. *NMR spectra of polymers and polymer additives*; CRC Press Taylor & Francis Group, 2000.
